# A cell cycle-coordinated Polymerase II transcription compartment encompasses gene expression before global genome activation

**DOI:** 10.1038/s41467-019-08487-5

**Published:** 2019-02-11

**Authors:** Yavor Hadzhiev, Haseeb K. Qureshi, Lucy Wheatley, Ledean Cooper, Aleksandra Jasiulewicz, Huy Van Nguyen, Joseph W. Wragg, Divyasree Poovathumkadavil, Sascha Conic, Sarah Bajan, Attila Sik, György Hutvàgner, Làszlò Tora, Agnieszka Gambus, John S. Fossey, Ferenc Müller

**Affiliations:** 1 Institute of Cancer and Genomic Sciences, College of Medical and Dental Sciences, University of Birmingham, Birmingham, B15 2TT UK; 2 School of Chemistry, University of Birmingham, Edgbaston, Birmingham, B15 2TT UK; 3 Institut de Génétique et de Biologie Moléculaire et Cellulaire, UMR7104, Centre National de la Recherche Scientifique, Strasbourg, 67404 Illkirch France; 4 Institut National de la Santé et de la Recherche Médicale, U964, Université de Strasbourg, Strasbourg, 67404 Illkirch France; 5 Faculty of Engineering and Information Technology, Biomedical Engineering School, University of Technology, Sydney, NSW 2007 Australia; 6 Institute of Transdisciplinary Discoveries, Institute of Physiology, Medical School, University of Pecs, Pecs, H-7624 Hungary; 7 Institute of Clinical Science, College of Medical and Dental Sciences, University of Birmingham, Birmingham, B15 2TT UK

## Abstract

Most metazoan embryos commence development with rapid, transcriptionally silent cell divisions, with genome activation delayed until the mid-blastula transition (MBT). However, a set of genes escapes global repression and gets activated before MBT. Here we describe the formation and the spatio-temporal dynamics of a pair of distinct transcription compartments, which encompasses the earliest gene expression in zebrafish. 4D imaging of pri-*miR430* and zinc-finger-gene activities by a novel, native transcription imaging approach reveals transcriptional sharing of nuclear compartments, which are regulated by homologous chromosome organisation. These compartments carry the majority of nascent-RNAs and active Polymerase II, are chromatin-depleted and represent the main sites of detectable transcription before MBT. Transcription occurs during the S-phase of increasingly permissive cleavage cycles. It is proposed, that the transcription compartment is part of the regulatory architecture of embryonic nuclei and offers a transcriptionally competent environment to facilitate early escape from repression before global genome activation.

## Abstract (web summary)

Transcription is globally repressed in early stage of embryo development, but a set of genes including pri-miR-430 and zinc finger genes is known to escape the repression. Here the authors image the very first transcriptional activities in the living zebra fish embryo, demonstrating a cell cycle-coordinated polymerase II transcription compartment.

## Introduction

Regulation of transcription underlies the coordination, determination and maintenance of cell identity, during organismal development. Nuclear topology and chromatin structure are key factors in the coordination of transcription of genes scattered across the genome (reviewed in ref .^1^). However, the relationship between spatio-temporal dynamics of transcription and the 4D organisation of the nucleus is poorly understood. Genome activation leads to the concerted activation of a large number of genes, which offers a tractable model to address the nuclear topology organisation of dynamic transcription. The earliest stages of development are under the exclusive control of maternally deposited proteins and RNAs, while the embryo remains in a transcriptionally silent state^2^. In externally developing metazoan embryos a series of extremely fast and metasynchronous cell division cycles precede global zygotic genome activation. Genome activation is regulated by a threshold nucleo-cytoplasmic (NC) ratio, which is reached at the mid-blastula transition (MBT)^3^ and reflects release from repression by diluted maternal factors, such as histones and replication factors^4–7^, Together with genome activation, simultaneous clearance of maternal RNAs^8^ at MBT overhauls the embryonic transcriptome^9^.

There is accumulating evidence for robust RNA Polymerase II (Pol II) transcription prior to global zygotic genome activation, in most animal models^2^. A small group of genes are activated several cell cycles before the MBT and represent the first wave of genome activation^10–12^. The first genes expressed in the zebrafish embryo include microRNAs, which drive the clearance of maternal mRNAs^13^, as well as transcription factors and chromatin binding proteins, which may play a role in the main wave of genome activation^10^. The existence of this first wave of genome activation raises the question of how genes escape the repressive environment before the threshold NC is reached at MBT. Furthermore, it remains unknown how transcription can occur during the short cell cycles consisting only of S and M-phases. To be able to address these questions, transcription monitoring at single-cell resolution is necessary, whilst maintaining the developing embryo context. This can only be achieved by in vivo imaging. Current imaging technologies are based on synthetic transgenic reporters of stem loop RNA-binding proteins, fused to fluorescent proteins^14^. This technology allowed monitoring of the dynamics, variation and nuclear topological constraints of gene expression (e.g.^15–18^.). However, its limitation is the requirement for transgenic manipulation of each gene of interest.

Here, native RNAs of endogenous genes were imaged, without the need to introduce fluorescent proteins by transgenesis. We developed a novel method based on arrays of fluorescently tagged antisense oligonucleotides^19^ for in vivo imaging of transcript accumulation and called it MOrpholino VIsualisation of Expression (MOVIE). MOVIE was used to demonstrate that the earliest gene expression in zebrafish embryos is confined to a unique transcription compartment, which forms during the S-phase of extremely short cleavage stage cell cycles, without gap phases. Nuclear organisation of transcription suggests a previously unappreciated nuclear body formation, which is seeded by transcribed loci on pairs of homologue chromosomes and persists in a cell cycle length-dependent manner. MOVIE enabled investigation of the spatio-temporal dynamics of this transcription compartment, which is a characteristic feature of pre-MBT embryos.

## Results

### MOVIE detects native gene transcription in living embryos

**Fig. 1:**
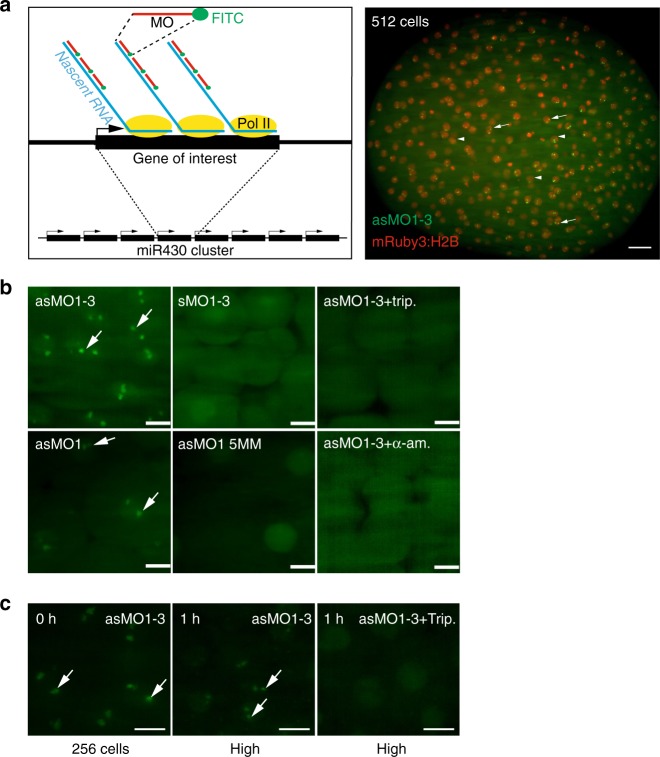
MOVIE detects native transcripts at the site of transcription in living zebrafish embryos. **a** Left: schematic of the detection principle of transcript imaging by MOVIE at the *miR430* primary miRNA gene cluster. Right: Image of whole embryo, imaged in vivo by lightsheet microscopy, injected with three antisense, FITC-labelled morpholinos (MO) targeting miR430 priRNA (green) and mRuby3:H2B fusion protein as nuclear marker (red). Arrows point at signal accumulation, appearing as double green punctae within the nuclei, labelled by mRuby3:H2B (arrowheads). **b** Representative images of live embryos, imaged in vivo at high/oblong by lightsheet microscopy, injected at zygote stage with triple and single sense, antisense and mismatch-containing, FITC-labelled morpholinos and transcription blockers (α-amanitin or triptolide, *n* = 6 embryos each). **c** Transcript signal detection before and after 1-hour triptolide treatment started at 256-cell stage (0 h), (n = 6 embryos each). Stages of embryos as indicated in panels. trip triptolide, as antisense, s sense, MO morpholino oligonucleotide (where number depicts specific MO, for details see Supplementary Table 1), 5 MM 5 mismatch containing, h hours after treatment. Scale bars: **a** 50 µm **b**, **c** 10 µm

**Fig. 2:**
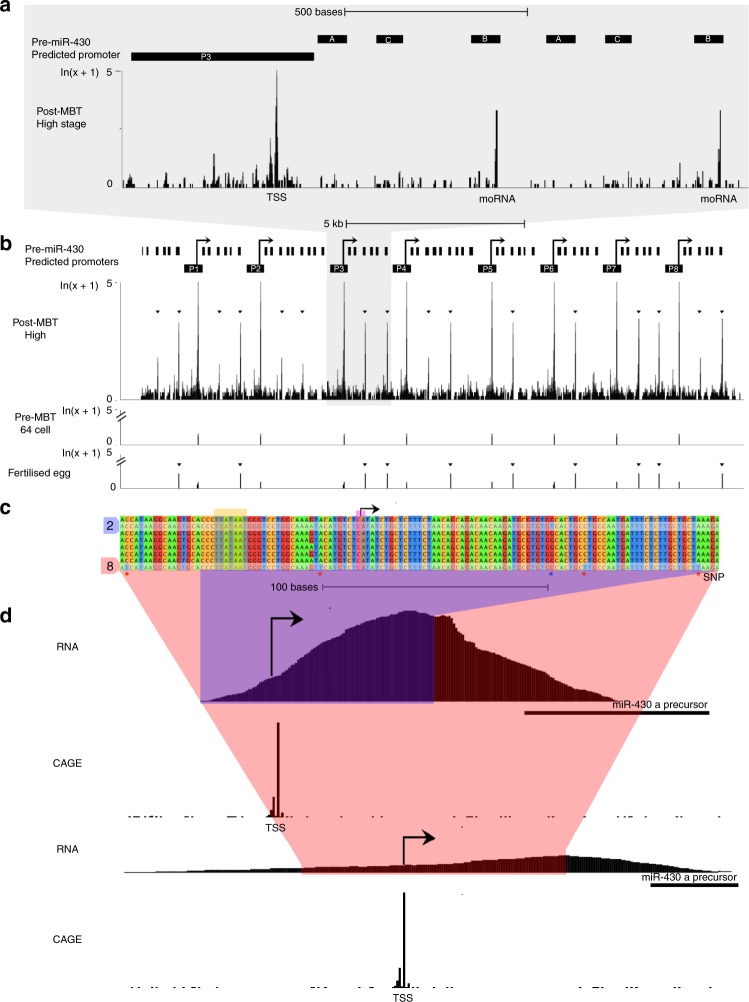
Characterisation of the *miR430* clustered locus demonstrates usage of multiple promoters. **a**, **b** Multi-site mapping of CAGE-seq provides evidence for activity of the miR430 cluster at early blastula stages. Schematic demonstrates transcription start sites (TSS) and internal RNA Drosha processing sites post (high stage) and pre-MBT (64-cell stage). The start position of microRNA offset RNA (moRNA) at the Drosha cutting site detected by CAGE is indicated. Black short bars indicate mature miRNA positions, long bar indicates predicted promoter region around the TSS. **b** At least 8 promoters are predicted by CAGE on the zebrafish reference genome (GRCz10). Candidate promoters are indicated by P1-P8, tag per million (TPM) signal is shown with a log scale. An additional cluster of miR430 genes without CAGE predicted promoter is shown on the left. **c** Promoter sequence alignment indicates SNPs among candidate promoters, which allow unique mapping of nascent RNA sequencing data for mapping of promoter usage. Orange box indicates predicted TATA box at the canonical position from the main TSS mapped by CAGE-seq. P3 and P9 carry unique bases for unique detection of transcripts driven by these promoters. **d** nascent RNA-seq data uniquely mapped to the miR430 locus demonstrates start site region utilization by at least two promoters indicated by CAGE-seq. RNA sequencing peaks drop at precursor gene start due to lack of SNP in the highly repetitive miRNA precursor region

We designed a morpholino oligonucleotide (MO) array and hypothesized that binding of morpholinos at 5′ ends of transcripts will lead to detectable fluorescence signal, due to their accumulation at endogenous gene loci, in living cells (Fig. 1a). We chose the primary transcripts of *miR430* microRNA genes as test for our approach. *MiR430* genes are the earliest and highest expressed genes during the first wave of zygotic gene activation^10^ and play a key role in the clearance of maternally deposited mRNA during MBT. Cap analysis of gene expression (CAGE) sequencing datasets^20^ were used to map the transcription start sites of the *miR430* gene cluster. CAGE data revealed at least eight transcriptional start sites on the reference zebrafish genome (GRCz10), with 6-9 *miR430* genes predicted to be transcribed from each of them (Fig. 2a, b). Two of these promoters contain single nucleotide polymorphisms (SNPs) allowing RNA-seq data to be uniquely mapped. This analysis demonstrated that at least two *miR430* promoters are used by the embryo (Fig. 2c, d). It was hypothesized that multiple promoter use at the locus may facilitate local enrichment of targeted MO fluorescence. A series of three *miR430*- targeting MOs (Supplementary Table 1) were generated, which upon microinjection into fertilised eggs led to the detection of two distinct transcription foci in nuclei of zebrafish blastula embryos, by lightsheet imaging (Fig. 1a, b). MO signal followed the developmental stages when *miR430* expression has previously been described^11,20^, (Supplementary Fig. 1a). Expression signals were detected as pairs of foci per nucleus, suggesting detection of miR430 RNAs from both parental alleles. The signal was RNA dependent, as indicated by lack of signal by sense MO, was specific to miR430 RNAs demonstrated by loss of signal with mismatch-containing MO and was lost upon transcription inhibition (Fig. 1b, c). We treated embryos with triptolide after detection of robust transcription at 256 cell stage (8^th^ cell-division) and found fluorescent foci were lost by high stage (10^th^ cell-division), indicating that transcripts produced earlier were not detectable in subsequent stages, in line with detection of newly transcribed RNAs by MOs (Fig. 1c). Additionally, MO injection did not affect miR430 RNA production or development of the embryos (Supplementary Fig. 1b,c). We thus demonstrated detection of native and nascent *miR430* primary RNA transcription in normally developing living vertebrate embryos by our novel method, which does not require transgenic manipulation.

### Transcription follows cleavage stage cell cycle periodicity

**Fig. 3:**
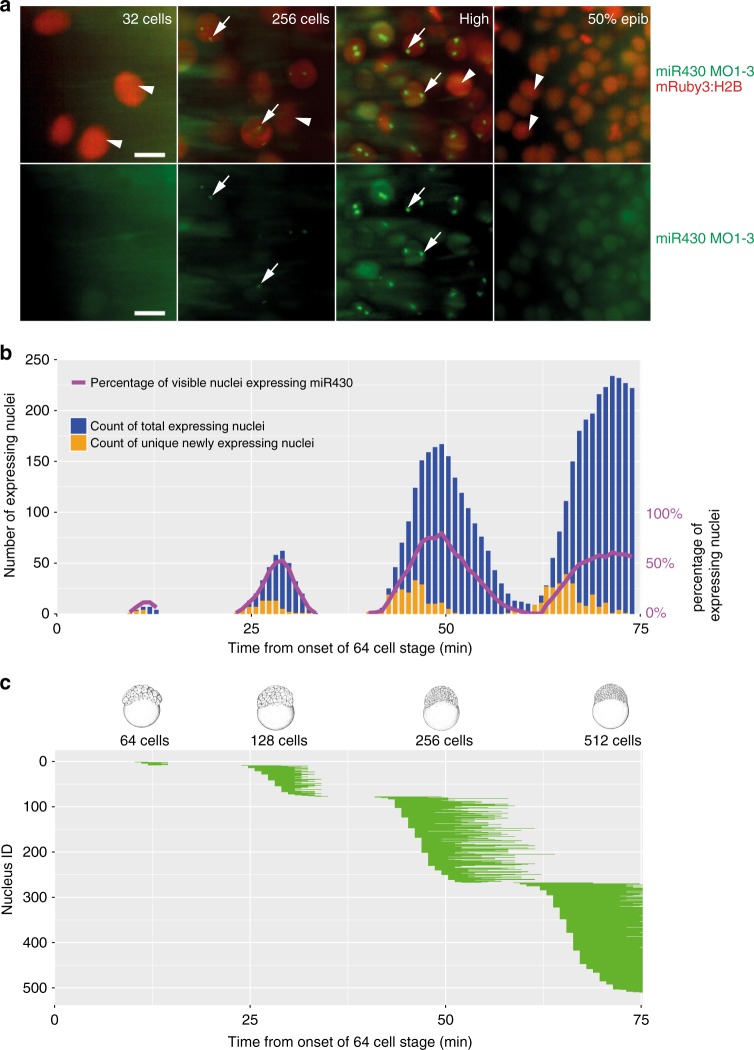
Mapping of gene expression dynamics at cellular resolution indicates that metasynchrony and periodicity follows the short cell cycle phases. **a** Representative examples of simultaneous detection of *miR430* expression by FITC-labelled MO in green (arrows) with mRuby3:H2B-labelled chromatin in red (arrowhead) during at stages as indicated. **b** Timing of the commencement of gene expression (orange) and number of nuclei (green) showing expression activity, total and new *miR430* expressing nuclei per frame. Stages of development are indicated at the bottom of the panels, purple line represent percentage of nuclei expressing *miR430* at the time and stages indicated. **c** Expression span of individual nuclei over time. Each horizontal bar represents the *miR430* expression time of a single nucleus. Chart *x*-axis matches that in (**b**). Each bar represents signal from up to 2 foci per nucleus. The frame rate (51 s) was used as reference for the elapsed time in (**b**, **c**). 50% epib, 50% epiboly. Scale bars: **a** 10 µm. Source data for (**b**, **c**) are provided as a Source Data file. Zebrafish embryonic stages schematics on panel (**b**) are reproduced from Kimmel et al., 1995, Developmental Dynamics 203:253-310 by permission of John Wiley & Sons, Inc. Source Data

Zebrafish cleavage stages are characterised by metasynchronous cell cycles^3^ in which synthesis and mitosis phases are cycling without gap phases^21–23^. To understand the temporal relationship between *miR430* transcription and the fast cell cycles of cleavage stages, we simultaneously monitored *miR430* expression by 4D time lapse imaging with MOVIE, and cell cleavages using the nuclear marker mRuby3:H2B (Fig. 3a, Supplementary Movie 1). Quantification of the miR430 transcript detection led to three main observations. Firstly, *miR430* expression was first detected at 64-cell stage, matching previous transcriptome analyses^10,20,24^, (Fig. 2a) and was present until late epiboly stages in diminishing numbers of cells. Secondly, the number of cells with *miR430* expression gradually increased during cleavage stages, potentially explaining the increase in genome-wide *miR430* expression quantification data from whole embryos (Fig. 3b, Supplementary Fig. 1a). By the 512-cell stage all detectable nuclei show *miR430* expression, however due to the loss of synchrony of this cell cycle the total number of cells expressing at any given time peaks at around 60% (Fig. 3c, Supplementary Fig. 2a). Thirdly, the temporal distribution of the start and extent of the MO signal showed gradually expanding asynchrony (Fig. 3c), which is in line with the previously described^25^ sequentially increasing length of pre-MBT cell cycles after the 6^th^ cell cycle (64-cell stage). Transcriptional activity in these cell cycles also followed a spatial wave-like pattern running across the animal pole (Supplementary Movie 2). There was no obvious allelic (parental) bias observed, with most cells showing two foci of activity. Thus transcription of *miR430* genes, first detected at the 64-cell stage, does not appear to respond to a precisely coordinated developmental stage timer. Instead, *miR430* expression appears in sequentially increasing number of nuclei and appears to be coordinated with the overall timing of cell cycles occurring with gradually increasing asynchrony.

### Transcription occurs in S-phase of elongating cell cycles

**Fig. 4:**
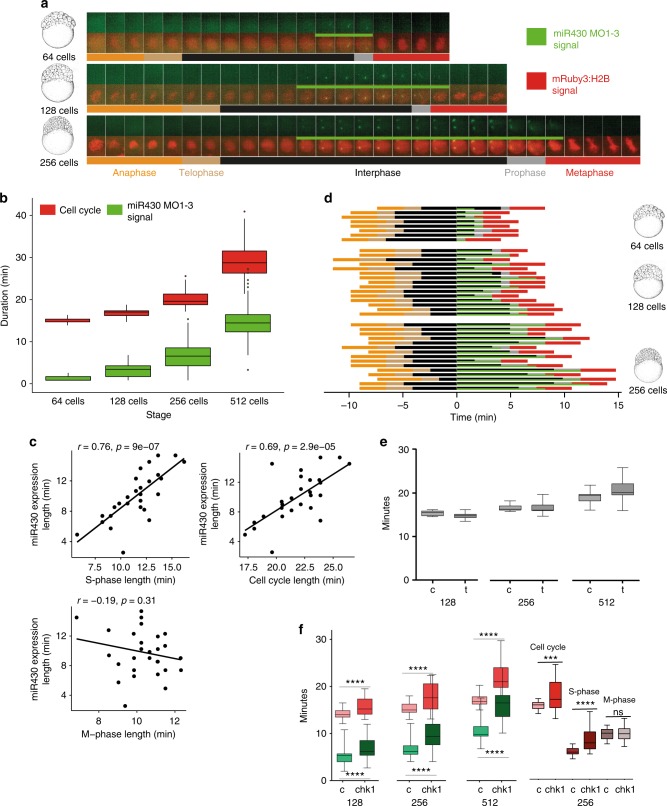
The S-phases of cleavage stage cell cycles are permissive to transcription and/or transcript retention. **a** Cell cycle length (anaphase to anaphase detected by mRuby3:H2B in red) and *miR430* expression (MO signal in green) during early blastula stages. Frames were acquired in 49 s intervals. **b** Box plot quantification of elongation of miR430 signal (green) alongside cell cycle length (red). Data from 2 embryos are calculated. Total number of nuclei (cell cycle): 64-cell = 17, 128-cell = 60, 256-cell = 62. All groups were significantly different from one another (*p* < 0.001) (One-way ANOVA with multiple comparisons). Middle line = median. whiskers = nearest hinge ± 1.5 * interquartile range. **c** Correlations of cell cycle, S-phase and M-phase length with *miR430* expression length, at 256-cell stage (2 embryos, 15 nuclei each). The Pearson correlation coefficient (r) and the corresponding *p*-value (p) are shown on each plot. **d** Cell cycle phase segmentation as shown in (**a**). Horizontal bars represent a nucleus each, they are aligned to onset of detection of *miR430* expression and ordered according to length of *miR430* expression and cell cycle length. *x*-axis shows time in minutes. **e** Effect of triptolide treatment on cleavage stage cell cycle length. Lightsheet microscopic images for nuclear pattern of mRuby3:H2B were analysed to assess cell cycle length and data merged from 4 embryos, 15 nuclei each. Water-injected controls (c) and triptolide-treated (t) are shown at stages as indicated. No statistical difference was found between c and t by Mann–Whitney *U* test. **f** Left: relationship between the lengths of cell cycle measured by mRuby3:H2B pattern (red) and pri-miR430 activity detection measured by MO signal (green) in control RNA and *chk1* mRNA injected embryos. Data was analysed from 4 embryos, 15 nuclei each at stages as indicated. Statistical significance was measured by Mann–Whitney *U* test, *p* < 0.001, whiskers show max and min values. The middle line represents the median. Right: comparison of cell cycle length, S-phase and M-phase for 256 cell stage nuclei, from the *chk1* injected and control embryos. Data from 3 embryos, cell cycles compared using Mann–Whitney test, and S and M-phase data compared using unpaired *t* test. ***P = 0.0004, ****P < 0.0001. Source data for (**b–f**) are provided as a Source Data file. Zebrafish embryonic stages schematics on panels (**a**, **d**) are reproduced from Kimmel et al., 1995, Developmental Dynamics 203:253-310 by permission of John Wiley & Sons, Inc. Source Data

The single-cell resolution of MOVIE allows the temporal dynamics of each cell’s transcriptional activity during metasynchronous cell cycles to be addressed. Tg(Xla.Eef1a1:h2b-mRFP1) transgenic embryos^26^ or mRuby3:H2B microinjected protein were used to analyse cell cycle length. Neither the red fluorescence-tagged H2B, nor MO injection affected the cell cycle (Supplementary Fig. 2b,c), allowing the accurate monitoring of the relationship between cell cycle and transcription. Cell cycle length was determined by measuring the time between two anaphases. Elongation of these cell cycles, which was previously demonstrated to be due the elongation of the S-phase^21^ was accompanied by elongation of miR430 signal detection (Fig. 4a, b). Cell cycle length varies among cells of a single cleavage stage^21^, therefore correlation between *miR430* expression length and cell cycle length was investigated among individual cells within a single cleavage stage. As shown in Fig. 4c, positive correlation was observed between both cell cycle length and S-phase length, but not mitosis length versus *miR430* expression. Next, we asked when *miR430* expression occurs within the alternating M and S-phases of the pre-MBT cell cycles. By using morphology distribution and entropy of mRuby3:H2B pixels as indicators for chromatin compaction, the cell cycle was segmented into its mitotic phases (Supplementary Fig. 2d) with the length of these phases measured (Fig. 4a, d). We detected the start of *miR430* activity universally during the S-phase of the pre-MBT cell cycles, but with varying delay (Fig. 4d), while the lowest levels of activity are potentially missed by the imaging approach.

An important observation from the cell cycle phase segmentation analysis was the detection of MO signals, albeit in reduced volume and intensity, into the prophase and even extending into metaphase, either suggesting transcription during M-phase or transcript retention at the transcription site (Fig. 4a, d, Supplementary Fig. 2e).

We have then asked whether transcription activity of mother cells has impact on transcription activity of daughter cells, which potentially indicate existence of transcription memory^15,27^. To this effect we clustered nuclei at 128 cell stage into expressing and non-expressing groups and then tracked their daughters at 256 and 512 cell stages. There was a statistically significant increase observed in both the on/off state and length of transcript detection in daughter cells that emerged from expressing mother cells (Supplementary Fig. 2f-h).

Spatio-temporal coupling of replication and transcription at the main wave of zygotic genome activation has previously been established^28–30^, yet this has not been explored during the little-studied first wave of genome activation. Our observation that the temporal extension of *miR430* activity matches that of the corresponding cell cycle lengthening, suggests the hypothesis that transcriptional activity is co-regulated with cell cycle elongation during cleavage stages. To test if transcription triggers cell cycle elongation at these early stages, transcription initiation was blocked by triptolide and its effect confirmed by the observation of loss of epiboly movements, as described previously^31^ (Supplementary Fig. 3a). No significant change was observed in cell cycle length, suggesting that cell cycle elongation in cleavage stage embryos is not explained by transcriptional activation (Fig. 4e). In contrast, when precocious Checkpoint kinase 1 (Chk1) activation was forced by injecting synthetic *chk1* mRNA^32,33^, significant elongation of the cell cycle in cleavage stage embryos and corresponding elongation of miR430 MO signal were detected in every cleavage cycle measured (Fig. 4f, Supplementary Fig. 3b,c). This elongation of the cell cycle was due to changes in S-phase as no significant changes were seen during M-phase (Fig. 4f). Thus, cell cycle elongation is permissive for *miR430* transcription and/or transcript retention in the sequentially expanding S-phases during cleavage stages in zebrafish.

### Transcription of first wave genes in a shared compartment

**Fig. 5:**
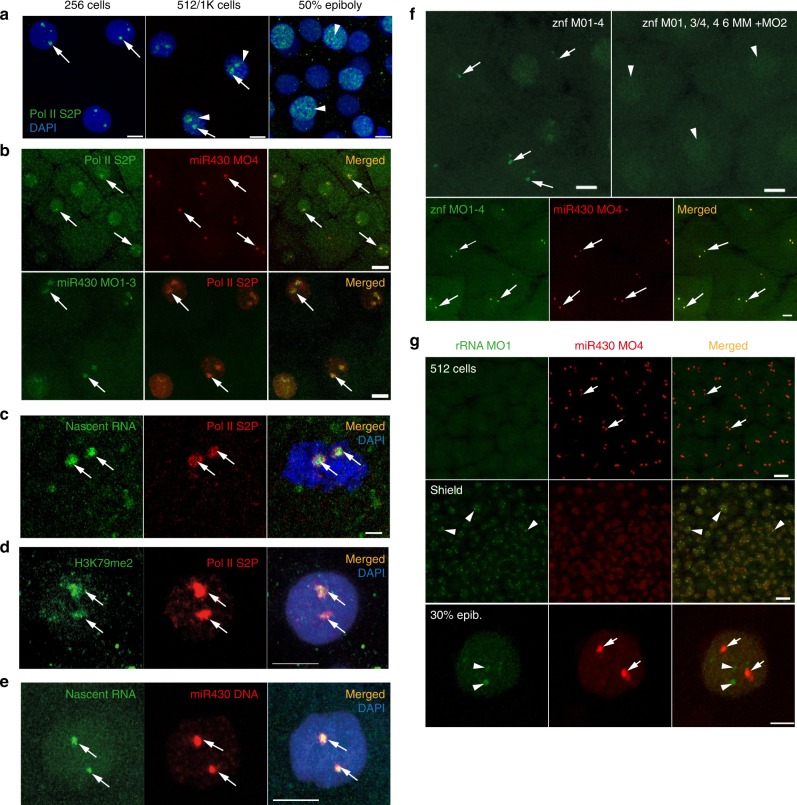
First wave transcription confined into a nuclear compartment. **a** Immunohistochemistry of Pol II S2P (green) with two main foci (arrows) and broader distribution (arrowheads) at indicated stages. Nuclei DAPI stained (blue). **b** Top: live imaging of embryos at 256-cell stage, injected with anti Pol II Ser2P Alexa Fluor 488 fab fragments (green) and Cy5-labelled miR430 MO4 (red). Green and red signals colocalise in all nuclei, with both signals detected (*n* = 3 embryos). Bottom: immunohistochemistry of Pol II S2P (red) and miR430 MO1-3 (green) at high stage; green and red signals colocalise in all nuclei (3 embryos). **c** Immunohistochemistry of Pol II S2P (red) and nascent RNA detection (green) by ethynyl-uridine (EU) incorporation and Alexa Fluor 488 tagging at 256-cell stage. Green and red signals colocalise in all nuclei (49 nuclei, 6 embryos). **d** Immunohistochemistry for H3K79me2 (green) and Pol II Ser2P (red) and merged image with DAPI (blue) of 512-cell stage embryos. Third panel shows merge with DAPI (8 nuclei, 3 embryos). **e** Staining for the *miR430* locus (red) in combination with nascent RNA detection (green) by EU at 512-cell stage. Merged panel shows DAPI in blue (*n* = 37 nuclei, 3 embryos). **f** Top panels: live detection of activity of a cluster of *znf* genes at 512-cell stage by fluorescein-labelled MOs, injected with mix of targeting (left) or mismatch-containing MOs (right) (*n* = 3 embryos each). Bottom panels: embryo co-injected with miR430 MO4 (red) and znf targeting MOs (green) at 256-cell stage. Green and red signals colocalised in all nuclei (*n* = 6 embryos). In panels (**b**–**f**) arrows point at transcription foci. **g** Top: Airyscan images of embryo (at 512-cell and shield stages) co-injected with MOs targeting the 5′ETS of 45 S ribosomal RNA precursor (green) and miR430 MO4 (red) (*n* = 4 embryos for each stage). Bottom: confocal Airyscan image of 30% epiboly embryo injected with the same reagents. miR430 MO4 signal accumulation (arrows) and rRNA_5’ETS_MO1 (arrowheads) are indicated. *znf* zinc finger gene, Pol II S2P RNA polymerase II serine-2 phosphorylated, rRNA ribosomal RNA, ETS external transcribed spacer, Scale bars (µm): **a** 10, **b** top 20, bottom 10, **c** 2, **d**, **e** 5, **f** 10, **g** top 20, bottom 5

The observation of a pair of pri-*miR430* foci raises the question of the nature and dynamics of this compartment in relation to other transcriptional activities, during the first wave of genome activation. An antibody that recognises phosphorylated serine-2 in the C-terminal domain (CTD) of the large subunit of RNA Polymerase II (Pol II Ser2P) was used as a marker of transcription elongation^34^. This revealed two large foci, similar to *miR430* in pre-MBT stages, that expand to broadly distributed irregular speckles after the main wave of global genome activation, likely reflecting the large expansion of transcription at thousands of genes^9,11^(Fig. 5a). Next, an in vivo approach was tested to simultaneously detect *mir430* and Pol II SerP2 activities. A single miR430 targeting MO, previously observed to be sufficient to detect *miR430* activity alone (Fig. 1b), was chosen for labelling with Cy5 (Supplementary Fig. 6), co-injected with labelled Fab fragments against Pol II Ser2P^35^ into embryos and imaged by confocal microscopy in vivo. Colocalisation was observed between Pol II Ser2P and miR430 MOs in live embryos, indicating that these newly described *miR430*-containing domains are transcriptionally highly active at pre-MBT stages (Fig. 5b) and appear to be the main site of Pol II activity at these stages. The colocalisation of Pol II SerP2 with miR430 transcripts was also verified by immunostaining (Fig. 5b). To further characterise cleavage stage transcription sites by an independent approach, a nucleotide analogue incorporation assay^36^ was used and nascent transcripts in pre-MBT embryos visualised with and without Pol II Ser2P localisation. This showed colocalisation of the majority of nascent RNAs together with Pol II Ser2P (Fig. 5c). Pol II Ser2P signal appeared as a dense accumulation of multiple speckles throughout the transcription compartment, marked by nascent RNA (Fig. 5c, Supplementary Fig. 5a, Supplementary Movie 7)

Furthermore, immunohistochemical detection of H3K79me2, an indicator of transcriptionally active chromatin^37^, at the site of Pol II Ser2P provides additional evidence for the majority of transcriptional activity being confined to two transcription compartments at these stages of development (Fig. 5d). A 200kbp BAC probe containing the *miR430* locus was detectable in nuclei as large, but distinct nuclear territories and colocalised with the main site of nascent RNA detection (Fig. 5e). This supports the suggestion that *miR430* activity marks the transcriptional compartment, which appears as the main site of early transcription and is characteristic of the first wave of genome activation.

Next, we asked whether expression of other genes, besides *miR430*, contribute to the formation of this transcription compartment. The expression of a partially annotated set of *zinc finger* (*znf)* genes, consisting of over 350 copies localised on the same chromosome as *mir430* (chr4)^11,38^ and peaking at main wave of ZGA^11^ were investigated during the first wave of genome activation using CAGE-seq data(Supplementary Fig. 4a). From these, 25 *znf* genes were found to be highly conserved (Supplementary Fig. 4b), and highly active at pre-MBT and scattered across a 37 Mbp region, immediately downstream of the *miR430* gene cluster on chromosome 4. The pre-MBT activity of *znf* genes suggested them as candidates for transcription visualisation and we designed MO sequences to the highly conserved 5′ end of their transcripts (Supplementary Fig. 4b, Supplementary Table 1). A series of quadruple MOs, which together target all 25 znfs (Supplementary Fig. 4c) were injected into embryos and led to specific detection of *znf* gene activity in two foci similar to *miR430* during cleavage and MBT stages (Fig. 5f). Using two-colour labelling of MOs for the simultaneous detection of fluorescein-tagged znf MOs and Cy5-tagged miR430 MO4, colocalisation of these two sets of RNAs, in a pair of shared transcription compartments, was demonstrated (Fig. 5f, Supplementary Movie 3).

Next the relationship of the observed transcription compartment with other compartment forming nuclear transcriptional activities was investigated. Four out of five loci of 45 S rDNA, which encode Pol I-transcribed ribosomal RNAs (rRNA) reside on chromosome 4^39^. Whether nucleoli forming rRNAs can also be visualised similarly to Pol II transcribed genes on the same chromosome was queried. Using MOVIE it was found that rRNA transcripts can be detected, however only after the main wave of genome activation at MBT, with low signals at 30% epiboly and frequent activity at shield stages (Fig. 5g), which is in line with previously described rRNA expression patterns^36,39^). At post-MBT stages, miR430 transcripts were still detected in a mosaic fashion (Fig. 5g) and allowed subnuclear localisation analysis of both RNAs when both gene products were detected in the same cells. Thus dual MO labelling permitted miR430 RNA and rRNA distribution to be compared. At 30% epiboly stage their accumulation was distinctly localised (Fig. 5g bottom row). This result demonstrates that the transcription compartment containing Pol II gene products is independent from the nucleolus-forming RNA compartment despite 4 of 5 annotated rRNA genes reside on chromosome 4.

### Transcription compartment and nuclear architecture

**Fig. 6:**
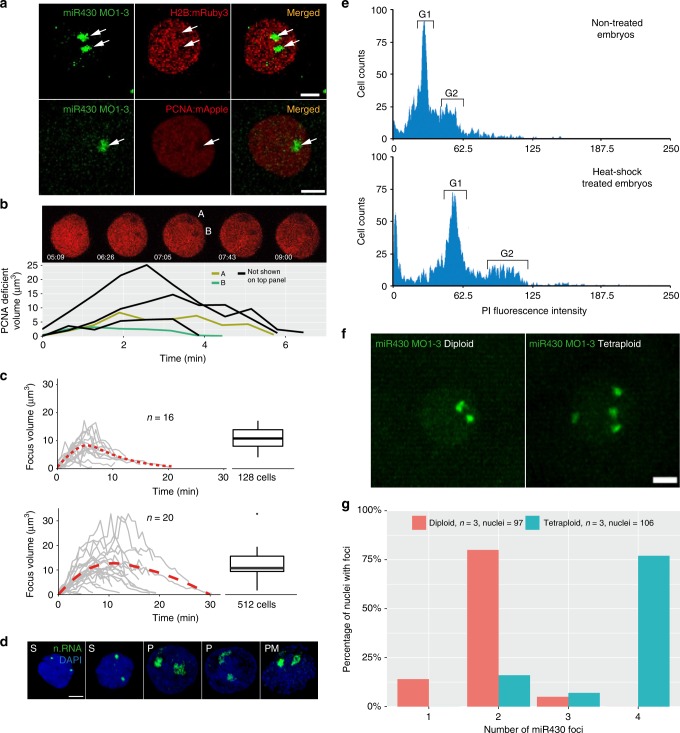
Relationship of the transcription compartment with the nuclear architecture. **a** Confocal Airyscan image of 512-cell stage embryos co-injected with miR430 MOs (green) and either mRuby3:H2B (top) or mApple:PCNA (bottom) fusion proteins (red). Arrows indicate low density chromatin at the site of *miR430* activity. **b** Time-lapse of 512-cell stage nucleus, injected with mApple:PCNA fusion proteins (red). Image shows individual z-slices from within the nucleus exhibiting two PCNA deficient regions. Graph below shows the evolution of the individual depicted PCNA deficient regions over time (A & B), in addition to deficient regions from other 512-cell stage nuclei from the same embryo, detected using spot detection plugin in Icy Bioimage. Time indicates duration from first detection of PCNA signal within the cell cycle in seconds and minutes. **c** Charts representing the transcription focus volume over time based on miR430 MO signal, measured using spot detection plugin Icy Bioimage software, top panel: 128-cell stage, bottom: 512-cell stage. Boxplots on the right show variation in peak volume. Middle line = median. whiskers = nearest hinge ± 1.5 * interquartile range. **d** Nascent RNA detection (green) by ethynyl-uridine incorporation and subsequent Alexa fluor 488 tagging of embryos fixed in 4 min intervals within 512-cells stage, demonstrating different stages of the transcription compartment structure during the cell cycle. **e** DNA content of 12-somite stage embryos untreated and heat-shocked was determined by propidium iodide DNA staining analysis. FACS traces show a shift in the position of the Gap phase 1 (G1) and Gap phase 2 (G2) peaks (marked), between the heat-shock untreated and treated groups, in accordance with a doubling in DNA content in the heat-shocked group. **f** Representative images of miR430 MO-injected diploid and induced polypoid 256-cell stage embryos (green). **g** Bar chart showing comparison of miR430 foci number between diploid and tetraploid embryos. n.RNA nascent RNA, S S-phase, P prophase, PM prometaphase; Scale bar, 5 µm. Source data for (**b**, **c**, **g**) are provided as a Source Data file. Source Data

Our results, obtained by several imaging tools, demonstrate the formation and prominence of a major transcription compartment, characterised by localised Pol II Ser2P enrichment and high concentration of newly synthesized nascent RNAs of several genes from at least two sets of gene families. Thus, it may fulfil several of the criteria for a nuclear body (reviewed in ref .^40^). Among them, local depletion of chromatin may occur, which has recently seen during formation of transcriptional microenvironments during the main wave of genome activation^41^. We therefore analysed chromatin state in pre-MBT embryos by simultaneous MOVIE imaging of miR430 RNA, together with mRuby3 labelled H2B and mApple labelled Proliferating Nuclear Antigen (PCNA), which is broadly localised to DNA in the nucleus during S-phase of cleavage stages in zebrafish^42^. Reduction of both chromatin-associated proteins was observed at the site of miR430 RNA accumulation and this depletion correlated with the dynamics of RNA detection (Fig. 6a). This lack of chromatin in a compartment was also seen without MO injection, or in embryos where endogenous Pol II Ser2P was detected without MO injection (Fig. 6b). Thus, we describe a nuclear transcription compartment, with coding and non-coding Pol II transcribed nascent RNAs, large quantities of transcriptionally active Pol II and local depletion of compact chromatin, which together suggest the formation of a transcription body in early embryos.

The temporal dynamics of the nuclear compartment in relation to cleavage stage cell cycles was addressed next. Measurement of miR430 MO signal and chromatin depletion volumes indicated highly dynamic formation, maintenance and disintegration of this compartment within a cell cycle (Fig. 6b, c, Supplementary Fig. 5b, Supplementary Movie 4). The transcription compartment has a characteristic volume dynamic, which shows gradual increase at both 128 and 512-cell stages peaking approximately at 16 μm^3^. At 512-cell stage the maximum volume shows an extended plateau in comparison to 128-cell stage, which correlates with the extended cell cycle (Fig. 6c, Supplementary Movie 5). The transcription compartment and its dynamics were also confirmed independently without MO injection, in fixed embryos, using nascent RNA detection by EU incorporation (Fig. 6d).

The presence of two transcription compartments in nuclei suggests that their formation is seeded by anti-paired^43^ homologue chromosomal loci occupying distinct chromosomal territories. To test the role of chromosomal organisation in forming transcription compartments, tetraploid embryos were generated by blocking centrosome separation during the first cell division using heat shock^44^ and their ploidy verified by FACS (Fig. 6e, f). The *miR430* activity foci were counted and it was found that the overall majority matched diploid allele count, whereas the number of foci was double that of diploid’s, in tetraploid embryos (Fig. 6g, Supplementary Movie 6). This finding suggests that the sites and number of transcription compartments are regulated by alleles on homologue chromosomes during cleavage stages. Interestingly, tetraploids, which have double the NC ratio and initiate MBT one cell cycle earlier than normal diploid wild-type embryos^3^, did not activate *miR430* expression earlier than diploids, which argues against a NC ratio threshold being the trigger for their gene expression before MBT (Supplementary Fig. 5c).

## Discussion

Here we describe a unique transcription compartment, the formation of which is characteristic to the first wave of gene expression in the embryo. Our results suggest that pre-MBT transcription escapes the global repressive effect of diluted maternal factors via the formation of a dynamic transcription compartment, where most detectable transcription from at least two gene families takes place. The observation that the majority of nascent RNAs and Pol II Ser2P colocalise with local depletion of chromatin, indicates the formation of a primordial nuclear body as the major site of detectable transcriptional activity, prior to global genome activation at MBT.

The temporal dynamics of this transcription compartment follows and is co-regulated with the metasynchronous cell divisions, both of which gradually elongate and increasingly lose synchrony at each cell cycle. This challenges long held views on MBT as a watershed stage; instead supporting a gradual process model for genome activation, previously proposed, based on gradually elongating cell cycles^45^. By manipulating transcription and cell cycle progression it was demonstrated herein that these sequentially expanding cell cycles are permissive for transcription and transcript accumulation and that the majority of transcriptional activity is during S-phase of the pre-MBT cell cycles, which lack gap phases^23,46^. Yet, it is unclear whether the observed presence of transcripts during prophase and prometaphase are remnants of nascent RNAs, or indicate de novo transcription. It is notable, that Pol II Ser2P is still present at prophase, potentially reflecting transcriptional activity in M-phase while it is unclear, whether this reflects elongating polymerase.

The mechanisms that allow pre-MBT transcription are likely different from that underlying the main wave of genome activation. Several mechanisms were suggested to underlie transcription activation in early embryos. These include a NC ratio threshold acting via titration of maternal factors^4^, a developmental timer acting via delayed access to transcriptional activators regulated by time-limited translation of maternal mRNAs (e.g^9^.), or availability of threshold amount of DNA template (reviewed in ref .^8,47^). In our experiments NC ratio does not appear to regulate the earliest of gene activation, as manipulation of nuclear content by inducing polyploidy did not have a marked effect on the timing of when expression is first detected. Furthermore, our observations on pre-MBT transcription detection at the single-cell level and with specific loci also exclude the model, which argues for lack of detectable transcription due to limiting amount of DNA template. Instead, our data suggests alternative mechanisms, which could include delayed availability of transcriptional activators, for example by the control of maternal mRNA translation.

The formation of the nuclear body may occur by several mechanisms. It is possible that the unique, repetitive organisation of the long arm of chromosome 4^11^ which includes the *miR430* cluster, as well as a series of *znf* genes together creates a local highly transcribing environment, which may function as an aggregation of transcription factories^48,49^ and appears as a distinguishable body. Interestingly, the *miR430* locus was shown to be specifically enriched for Rad21 binding before zygotic genome activation^50^, which suggests the involvement of cohesins in the formation of a distinct local chromatin environment. Additional contributing factors to the formation of the large compartment may involve phase separation principles^40,41^ or other means of local accumulation of RNA at the transcription site. Nevertheless, by detection of specific chromosomal loci and their RNA products, as well as by ploidy manipulation, we demonstrated that these nuclear bodies are seeded by DNA and that the candidate loci for coordinating these nuclear compartments reside on homologues of chromosome 4 occupying distinct territories in the nucleus. While our rRNA imaging data suggests separation of Pol II and nucleolus-forming rRNA compartments, future studies will be necessary to investigate the relationship between the transcription compartment described here and other nuclear bodies, such as Cajal body and the histone locus body^36^ during early zebrafish development.

The formation of the transcription compartments may serve several purposes in the uniquely fast cell division cycles that precede global genome activation. Firstly, it may allow for efficient transcription by concentrating transcription apparatus into a confined nuclear space, at a time when activators and transcription machinery components are likely rate limiting due to delayed translation of maternal stockpiles of their mRNAs^24,51^. Secondly, restricted localisation of transcription into a compartment may indicate an as yet unexplored mechanism for facilitating the escape from the general repressive environment of the nucleus, which is only removed from the rest of the genome by reaching a certain NC ratio. Initial observations however, suggest that the first wave of transcription is not affected by similar titration of maternal repressive factors as the rest of the genome, as indicated by unaffected initiation of transcription in tetraploids, where the NC ratio is doubled. Thirdly, it may provide spatial and temporal separation of replication and transcription—two potentially conflicting genome activities occurring in the short S-phases without gap^52^. The transcription compartment with activities of genes residing on chromosome 4 raises the question of where first wave-expressed genes residing on other chromosomes are transcribed in the pre-MBT nucleus. While most Pol II Ser2 and nascent RNAs are detected to colocalise with the pri-miR430 signal, it is not excluded that low expressing loci are active in a separate domains from the chromosome 4 territory. Future work aiming to improve detection of low expressed single copy gene products will provide insight into the generality of the transcription compartment described in this study.

The compartmentalisation of the first wave of genome activation is likely a general phenomenon and not unique to zebrafish. In situ detection of large Pol II Ser2P and Pol II pSer5 punctae in *Drosophila* embryos during and before global genome activation, has been documented^53–56^ and may explain the first wave of genome activation not only in *Drosophila*, but more generally among externally developing animal models. Due to the simplicity of the MOVIE approach, testing this hypothesis in early embryos of other animal models will be feasible. Utilising multicopy gene product detection, MOVIE will be particularly useful to study RNA accumulation in nuclear bodies. Taken together, our in vivo monitoring of nuclear architecture organisation into transcription compartments contributes to the growing body of evidence supporting the notion of suprachromosomal organisation in the nucleus, serving not only structural, but also gene regulatory function^57,58^ and highlights the early embryo as a tractable model system to study the interface between transcription and nuclear organisation.

## Methods

### Characterisation of *miR430* locus

Identification of predicted multiple promoter sites at the *miR430* locus were determined from CAGE-seq data^20^ mapped to zebrafish assembly GRCz10 by Tophat^59^ allowing up to 80 multi-mapped reads. Start sites of transcription were determined by usage of CAGEr software^60^. Annotation of promoter regions (Fig. 2) were defined as 400 bases upstream and 100 bases downstream of the detected TSS with pre-*miR430* gene annotations determined from Ensembl database with addition of 2 pre-*miR430* c-subtype sequences found by BLAT search, completing the a-c-b triplet structure^61^.

Detection of unique SNPs in *miR430* promoter sequences was achieved by multiple sequence alignment with Clustal Omega^62^. Thus allowing detection of nascent RNA^10^ reads aligning to two individual promoters in the *miR430* cluster by unique mapping with STAR aligner^63^. Expression of *miR430* during development is demonstrated both by CAGE-seq^20^ and RNA-seq^11^ spanning 19 developmental time points, capturing expression both pre and post-MBT (Supplementary Fig. 1a).

### Morpholino design for *ZNF* genes on chromosome 4

Targeting MOs were designed specifically to visualise transcription of zinc finger genes on chromosome 4, most highly expressed in the early zygote, as determined from CAGE-seq dataset^20^. Twenty three such genes with a fully annotated transcript were identified. The 5′ UTRs of these genes with 50 bp up- and downstream flanking sequence were used for conservation assessment, via multiple sequence alignment using MUSCLE alignment algorithm^64^ and subsequent visualizations of inherent similarity were achieved with JalView^65^. Consensus sequences were generated and used for morpholino design, targeting conserved regions in the 5′UTRs. Altogether 4 morpholino were designed (Supplementary Fig. 4b).

To identify all potential *znf* genes on chromosome 4, targeted by the designed MOs, a BLAST search against Ensembl version 92 cDNA database was performed. A transcript was considered as a potential target by a morpholino if at least a 20 bp match with 84% identity was found. Intersections were calculated from the identified potential target genes list for each morpholino by VennMaster^66^ and quad venn-diagram graphic (Supplementary Fig. 4c) was generated with VennDiagram CRAN R package^67^.

### Synthesis of Cy5-tagged miR430 MO4 morpholino

Unless otherwise stated materials and reagent were used as supplied by Sigma-Aldrich and Fisher Scientific. Morpholino sequence (I) bearing 3′-alkyne functionality attached by propyl acetate linker were obtained from Gene Tools Ltd and used as supplied. Desalting was conducted using NAP-10 columns (GE Healthcare). HPLC analysis and purification was carried out using a Shimadzu LC2010 with a Phenomenex Kinetex 5 µm C18 100 Å (250 × 4.6 mm) column. Mass spectrometry was conducted on a SYNAPT G2-S instrument using TOF MS ES + with a solvent mixture of 50:50 water/acetonitrile containing formic acid (0.1% v/v). UV absorption spectroscopy was conducted on Shimadzu UV-180 UV spectrophotometer. Solvents used were of HPLC grade, Milli-Q water is ultrapure water supplied by Millipore Corporation.

An alkyne-tagged miR430 MO4 sequence (I) was exposed to azide-tagged Cy5-fluorophore II under copper-catalysed triazole-forming conditions to deliver Cy5-tagged sequence III. Fluorescein-tagged morpholino sequence IV (and alkyne-tagged sequence I) were supplied by Gene Tools Ltd. The conversion to III is detailed in Supplementary Fig. 6a.

Tris(3-hydroxypropyltriazolylmethyl)amine (THPTA) (11 mg, 25.5 µmol), sodium abscorbate (5.3 mg, 27 µmol) copper sulphate (0.94 mg, 3.75 µmol) and a magnetic stirring bead were placed in a 2 mL vial. 3′-Alkyne morpholino sequence (I, 150 nmol, from Gene Tools) was dissolved in water (500 µL, milli-Q) and was transferred to the reaction vial. Cy5 azide (II, 225 nmol) dissolved in water (250 µL, milli-Q) was added to the reaction mixture. A further addition of water (250 µL, milli-Q) brought the total volume of the mixture to 1 mL. The mixture was stirred at room temperature for 16 h. The reaction mixture was desalted by passing through a NAP column and rinsed with additional water (0.5 mL, milli- Q) to obtained 1.5 mL of product-containing mixture.

The Cy5-tagged oligo miR430 MO4 was purified by analytical scale HPLC using conditions as follows:

Phenomenex Kinetex 5 µm C18 100 Å (250 × 4.6 mm) as the stationary phase and a defined mixture of water and acetonitrile as the mobile phase.

Flow rate: 2 mL/min

Column temperature: 35 °C

Gradient:

- (i) 95:5 water:acetonitrile (0 - > 3.5 min)
- (ii) 70:30 water:acetonitrile (3.5 - > 8.5 min)
- (iii) 0:100 water:acetonitrile (8.5 - > 13 min)
- (iv) 95:5 water:acetonitrile (0 - > 3.5 min)

Detection: UV/vis: 254, 260, 647 and 663 nm; Excitation 647 nm Emission 633 nm

*Collection*: A peak in the chromatogram centred at 6.1 min was collected. The peak thus described displayed absorbances at the four detection wavelengths and fluorescence under the excitation and emission conditions described. The material collected was freeze dried and re-dissolved water (100 µL, milli-Q). Concentration: The concentration was determined by UV-Vis absorption, using absorption equation *A* = *ε.l.c* (where *A* = absorption, *l* is the path length, *c* = concentration and the extinction coefficient (*ε*) of the Cy5 *ε*650 = 250000 M^−1^cm^−1^). The collected fraction was diluted (20 μL of sample fraction and 140 μL of water) to 160 μL of analyte solution with an observed absorption of 0.81084. Path length was 1 cm. The calculated concentration was thus determined to be 25.9 μM. The resulting solution was analysed for purity by analytical HPLC, using a Phenomenex Kinetex 5 µm C18 100 Å (250 × 4.6 mm).

Flow rate: 1 mL/min

Column temperature: 35 °C

Gradient:

- (i) 95:5 water:acetonitrile (0 - > 25 min)
- (ii) 82:18 water:acetonitrile (25 - > 30 min)
- (iii) 0:100 water:acetonitrile (30 - > 40 min)
- (iv) 95:5 water:acetonitrile (40 - > 45 min)

Detection: UV/vis 260 and 645 nm 

A single peak in the chromatogram centered at 29 mins displayed absorbance at both 260 and 645 nm (Supplementary Fig. 6b,c) and had the correct m/z ([M + ] III) of 9258 by mass spectrometry (Supplementary Fig. 6d,e,f).

### Antibody Fab fragments production and labelling

The mouse monoclonal antibody against human RPB1 CTD, phosphorylated at serine-2 (MABI0602) was purchased from MBL laboratories (Nagoya, Japan). Fab fragments were prepared by digesting the antibodies with papain. A total amount of 200 μg of antibodies were reduced by adding 1 mM Tris(2-carboxyethyl)phosphine (C4706, Sigma-Aldrich, St. Louis, MO) and afterwards digested using 100 μl of magnetic beads coated with papain (PAPM-40-2, Spherotech Inc., Libertyville, IL) for 3 h at 37 °C. The Fab fragments were subsequently separated from Fc fragments by purification on 100 μl of Protein A Sepharose beads (GE Healthcare, Issaquah, WA) for 30 min at 4 °C. Afterwards, 100 μg of Fab fragments were fluorescently labelled using an Alexa Fluor 568 Monoclonal Antibody Labelling Kit (A20184, Thermo Fisher, Waltham, MA) following the manufacturers protocol.

### Zebrafish maintenance

All zebrafish strains were maintained in designated facility (according to UK Home Office regulations) in a recirculating system (ZebTEC, Tecniplast) at 26 °C in a 10-hour dark, 14-hour light photoperiod and fed three times daily.

Zebrafish experiments were restricted to early embryos and adults were only used for natural breeding. Animal work presented in this study was carried out under the project licences 40/3681 and P51AB7F76 assigned to the University of Birmingham, UK

### Microinjection

Single-cell stage embryos were injected with 2 nl solutions containing targeting or mismatch control morpholinos at a concentration of 7 µM each in nuclease-free water, supplemented with 0.1% phenol red. Unlabelled and fluorescein-labelled morpholinos were ordered from Gene Tools LLC.

At the low concentration used, MOs did not cause developmental defects in the majority of embryos, and did not affect mature miRNA processing from the primary transcripts (Supplementary Fig. 1b,c). Upon co-injection with mRuby3:H2B, mApple:PCNA fusion proteins or antibody, the final concentration of proteins in the injection solution was 400 ng/µl for the fusion proteins and 250 ng/µl for the antibody. We verified that neither recombinant H2B protein nor injection of MO affected the cell cycle (c,c). After injection the embryos were kept in E3 media at 28 °C until imaging or fixation.

### Transcription block

Transcription block was performed by treatment with 1 µM triptolide from the single-cell stage (Sigma T3652) in E3 media, or by microinjection of 200 pg α-amanitin (Sigma A2263) at single-cell stage.

### Live embryo imaging

Imaging of live embryos injected with fluorescent labelled MOs, fluorescent protein fusions or antibodies was performed on Zeiss Lightsheet Z1 or Zeiss 880 confocal microscope with Fast Airyscan Module.

For imaging on Zeiss Lightsheet Z1, embryos were mounted in 1% low melt agarose column using size three glass capillaries and incubated in E3 media at 28 °C during imaging. Z-stacks of ~200–300 slices in 0.5–1 µm steps were acquired every 30–60 seconds for 1.5–2.5 h, with ×20 objective.

For imaging on the Zeiss 880 confocal microscope in Fast Airyscan mode, embryos were mounted in 0.5% low melting agarose in glass bottom imaging dish and incubation chamber temperature was maintained at 28 °C during imaging. Z-stacks of ~100–200 slices were acquired (step size of 0.25 µm) every 30–60 seconds for 1.5–2.5 h using a ×25 or ×40 objectives. Post-processing of the acquired images was performed using Zeiss ZEN Blue Software.

### Analysis of spatio-temporal dynamics of MO signal

For global temporal analyses, lightsheet microscopy (morpholino/transgenic mRuby3:H2B) datasets were transformed into maximum intensity projections (MIPs), using Zeiss Zen Black software. Each MIP was imported into Icy Bioimage (versions 1.9.5.0–1.9.7.0, Quantitative Image Analysis Unit, Pasteur Institute, France), where the spot detection plugin was used to detect transcription foci and nuclei, as these features are represented as regions of locally elevated intensity, in their, respective, channels. The plugin was guided to detect features as bright regions against a darker background. Transcription foci were typically detected using a pixel size parameter of 3px, with a sensitivity of between 80 and 150% depending on noise (with higher sensitivity for higher noise). Feature size limits for detecting transcription foci were between 3 and 75 pixels in area. Nuclei were detected using a pixel size of between 13 and 25px, with similar sensitivity settings. Feature size limits for detecting nuclei were between 200 and 3000 pixels in area. Spot detection produced 2D regions of interest (ROIs) around features for each channel separately. Transcription foci were given a label which associated with their corresponding nucleus, and this was applied on a frame by frame basis to allow counting of nuclei expressing transcription foci. ROI metadata was exported to Microsoft Excel where transcription foci could be quantified.

### 3D analysis

3D datasets (Airyscan morpholino/injected mRuby3:H2B protein) collected from the Zeiss 880 confocal microscope with Fast Airyscan Module were imported into Icy Bioimage, where the spot detection plugin was used to identify nuclei and transcription foci, using the same sensitivity settings as described previously. Size features were scaled up to account for volumes: feature size settings for transcription foci were between 5 and 150 pixels, and size settings for nuclei were between 750 and 20,000 pixels. This would produce a 3D ROI around the detected feature. In the case of nuclei this ROI could be viewed in the 3D VTK viewer on Icy Bioimage, where a video of the rendered ROI could be recorded using the built-in recording function (Supplementary Fig. 5). ROI data were exported to Microsoft Excel where transcription foci volumes were extracted per frame and timeseries could be plotted (Fig. 6c).

In PCNA imaging data obtained on Zeiss 880 confocal with Airyscan, PCNA deficient regions were quantified using the spot detection plugin, with the setting to search for dark features against a bright background. Pixel sensitivity and 3D size settings were the same as for transcription foci detection, described previously. Relevant ROI’s were confirmed by visually inspecting that they correlated with signal deficient regions, and labelling the same feature with the same name per frame. The volume for each feature was extracted and plotted over time using Microsoft Excel.

### Cell cycle segmentation reference

Cell cycle phase segmentation was based on a visual library of cell cycle phase features generated from previously published confocal imaging of chromatin^68–70^. This reference library was used as a base for segmenting nuclei from MIP Zeiss Lightsheet data into cell cycle phases. Once phases were assigned to an initial batch of nuclei, the imaging parameters were extracted. These parameters were used to generate a quantitative measure of chromatin consistency throughout the cell cycle and assign a measure of cell cycle phase to each nucleus. Imaging parameters included 2D area covered by a nucleus ROI and standard deviation in pixel intensities across the nucleus (Supplementary Fig. 2d).

### Whole mount antibody staining

Embryos were fixed at the desired stage in 4% PFA in PBS then permeabilised with PBS-0.3% TritonX-100. Blocking was performed with room temperature blocking solution (BlockAid™, B10710, Thermo Fisher) then transferred to blocking solution containing primary antibody. Excess primary antibody was removed with PBS-0.1% Tween-20 washes. Samples were then incubated with blocking solution containing appropriate secondary antibody conjugated to Alexa Fluor fluorescent dye at 1:500 dilution. Immunostained embryos were imaged in antifade mounting media with DAPI (Vectashield, H-1200, Vector Laboratories) on Zeiss 880 confocal microscope with Fast Airyscan Module.

Primary antibodies used:

- Anti-RNA polymerase II (phospho S2), ab5095, Abcam at 1:400 dilution
- Pol II S2P monoclonal antibody, C15200005, Diagenode at 1:1000 dilution
- Anti-Histone H3 (di methyl K79) antibody, ab3594, Abcam at 1:1000 dilution

### Nascent RNA staining

Zebrafish embryos were injected with 1 nl of 50 mM ethynyl-uridine (EU, Thermo Fisher, C10329) solution at single-cell stage, incubated in E3 media at 28 °C until fixation at the desired stage. Embryos were permeabilised with PBS-0.3% TritonX-100 before undergoing detection of nascent RNA using the Click-iT™ RNA Alexa Fluor™ 488 Imaging Kit (Thermo Fisher, C10329), following the manufacturer’s protocol. After the reaction embryos were imaged on Zeiss 880 confocal microscope with Fast Airyscan, or used for subsequent protein or DNA detection.

### DNA FISH

Probes for DNA-FISH were prepared using the FISH Tag™ DNA Multicolor Kit (Thermo Fisher) following kit instructions. The BAC DKEY-69C19 was used as a template for DNA-FISH probe production, corresponding to 214 kb of the *mir430* locus on chromosome 4.

Embryos were fixed at the developmental stage of interest using 4% PFA. Animal caps were isolated and a series of gradient washes was used to equilibrate the samples with hybridization buffer [50% formamide, 4x SSC, 100 mM NaPO4 pH 7.0, 0.1% Tween-20]. Embryonic DNA was denatured by incubation of samples at 70 °C for 15 min before application of probe and incubation overnight at 37 °C. Unbound probe was washed from the samples using a hybridization buffer: PBS-T gradient. Samples were counterstained with DAPI prior to imaging on a Zeiss LSM 880 with Fast Airyscan, with a 100 × 1.48 numerical aperture objective lens. 50–100 optical sections (130 nm thickness) were acquired of each nucleus. Acquired images were processed using Zen Black software (Zeiss).

### Transgenic lines

The transgenic line Tg(Xla.Eef1a1:h2b-mRFP1) (http://zfin.org/ZDB-TGCONSTRCT-130711-1^26^) used in this study was kindly provided by Sarah Baxendale (University of Sheffield, UK).

### Protein synthesis

mRuby3:H2B and mApple:PCNA were PCR amplified form plasmids *pKanCMV-mRuby3-10aa-H2B* (Addgene, 74258) and *mApple-PCNA-19-NLS-4* (Addgene, 54937), respectively, and sub-cloned into NdeI/BamHI linearized *pET-28a* *+* plasmid (in frame with the N-terminal 6xHis-Tag), using In-Fusion® HD Cloning Plus Kit (Clontech, 638910) following the manufacture instructions.

The plasmids were transformed into *E. coli* BL21 (DE3) and the proteins were expressed in LB media by IPTG induction at 20°C. The cell lysates containing mApple:PCNA were prepared by re-suspending in 50 mM Tris HCl; pH 8, 250 mM NaCl, 10 mM imidazole, 2 mM MgCl2 and 10% glycerol whereas mRuby3:H2B was resuspended in 50 mM Tris HCl; pH 8, 500 mM NaCl, 10 mM imidazole, 2 m M MgCl2, 5% glycerol and 0.3% Brij. All buffers were additionally supplemented with BitNuclease, PMSF and protease inhibitors. Cell lysis was improved by subjecting the lysates to sonication followed by centrifugation to clarify the cell extracts. The proteins were then purified by Nickel affinity chromatography (Generon). The proteins were dialysed against 500 mM KCl, 20 mM PIPES, 100 μM EGTA, pH6.8 using 12 kDa columns (Pur-A-Lyzer; Sigma).

### Manipulation of embryonic cell ploidy by heat-shock

Tetraploid embryos were generated by heat-shock treatment, following the HS2 protocol adapted from^44^. Embryos were collected 2 min post fertilization and maintained in E3 media for a further 20 min at 28 °C, during which microinjection of MOs was performed. Embryos were heat-shocked between 20 and 23 min post fertilization by incubation in E3 media at 42 °C for 2 min, then returned to E3 media at 28 °C and allowed to develop normally. Embryos were monitored at the normal point of the second cell division (1 h post fertilisation (hpf)) and heat-shock treated embryos that failed to undergo cytokinesis were selected for downstream analysis, along with matched non-heat-shock treated controls. For the assessment of ploidy manipulation, embryos were collected at the 12-somite stage, dissociated with PBS-based enzyme-free cell dissociation buffer (Gibco), washed with PBS and subjected to propidium iodide DNA content analysis following manufacturer’s conditions (Invitrogen). Flow cytometry was performed on the CyAn ADP machine (Beckman Coulter) using the Summit 4.4 software package using the standard gating strategy.

### Cell cycle length and gene expression experiments

Wild type embryos were injected with mRuby3:H2B protein at the single-cell stage and incubated in E3 media supplemented with 1 µM triptolide (Sigma, T3652) or vehicle control following injection. In cell cycle elongation experiments, wild-type embryos were injected at the single-cell stage with a cocktail of 200 pg of *Xenopus chk1* mRNA, 200 pg miR430 MO and mRuby3:H2B protein whereas controls were injected with 200 pg *mCherry* mRNA + 200 pg miR430 MO and mRuby3:H2B protein. Embryos were imaged using Zeiss Lightsheet Z.1 microscope with the ZEN software. Images were analysed and timing of cell cycle transition from anaphase to anaphase were identified by mRuby3:H2B pattern variation using Icy Bioimage. Statistical analysis was carried out by Prism GraphPad 5 (USA). *Chk1* and *mCherry* mRNAs were produced from *NotI* linearized *pCS2* *+* *:chk1* (kindly provided by Phil Zegerman) and *pCS2* *+* *:mCherry* vectors, respectively, using mMESSAGE mMACHINE SP6 Transcription Kit (ThermoFisher Scientific).

### Quantification of miRNA levels by digital droplet PCR

Total RNA was extracted from a 100 miR430 targeting MO1-3 and non-injected embryos at sphere stage, using TRIzol™ Reagent (ThermoFisher, 15596026) following manufacturer’s instructions. Three RNA samples for each group (MOs injected and non-injected) from three independent experiments were prepared. cDNA was synthesized from equal amounts of total RNA (100 ng) using the High-Capacity cDNA Reverse Transcription Kit (Applied Biosystems) following the manufacturer’s protocol. cDNA samples were diluted 1:30 with nuclease-free water. qPCR reactions were prepared using the ddSupermix for Probes (Bio-rad) and Taqman miRNA probes miR430a (004365_mat) and miR430b (465767_mat). Droplet generation oil was added to the qPCR mixture and droplets were generated with the QX100 droplet generator (Bio-rad). PCR protocol as per manufacturer (Taqman miRNA assay) and the samples were run in a QX100 droplet reader. Quantasoft software (Bio-rad) was used for data acquisition and analysis.

### Reporting summary

Further information on experimental design is available in the Nature Research Reporting Summary linked to this article.

## Supplementary information

Supplemetary Information

Description of Additional Supplementary Information

Supplementary Movie 1

Supplementary Movie 2

Supplementary Movie 3

Supplementary Movie 4

Supplementary Movie 5

Supplementary Movie 6

Supplementary Movie 7

Reporting Summary

Source Data

## Data Availability

The source data underlying Figs. 3b,c; 4b–f; 6b,c,g and Supplementary Fig. 1a-c; 2a-d,f-g; 3b,c; 4c; 5b are provided as a Source Data File. All 3D, 4D imaging data are available upon request. A reporting summary for this Article is available as a Supplementary Information file.
