# Supplementary material for: A cell cycle-coordinated Polymerase II transcription compartment encompasses gene expression before global genome activation
Source: Nat Commun. 2019 Feb 11;10:691. doi: 10.1038/s41467-019-08487-5 (PMC6370886; doi:10.1038/s41467-019-08487-5)
Supplement: Supplementary file 1 — Supplemetary Information [file 41467_2019_8487_MOESM1_ESM.pdf]

A cell cycle-coordinated Polymerase II transcription compartment encompasses gene expression before global genome activation

Hadzhiev et al.

Supplementary Information

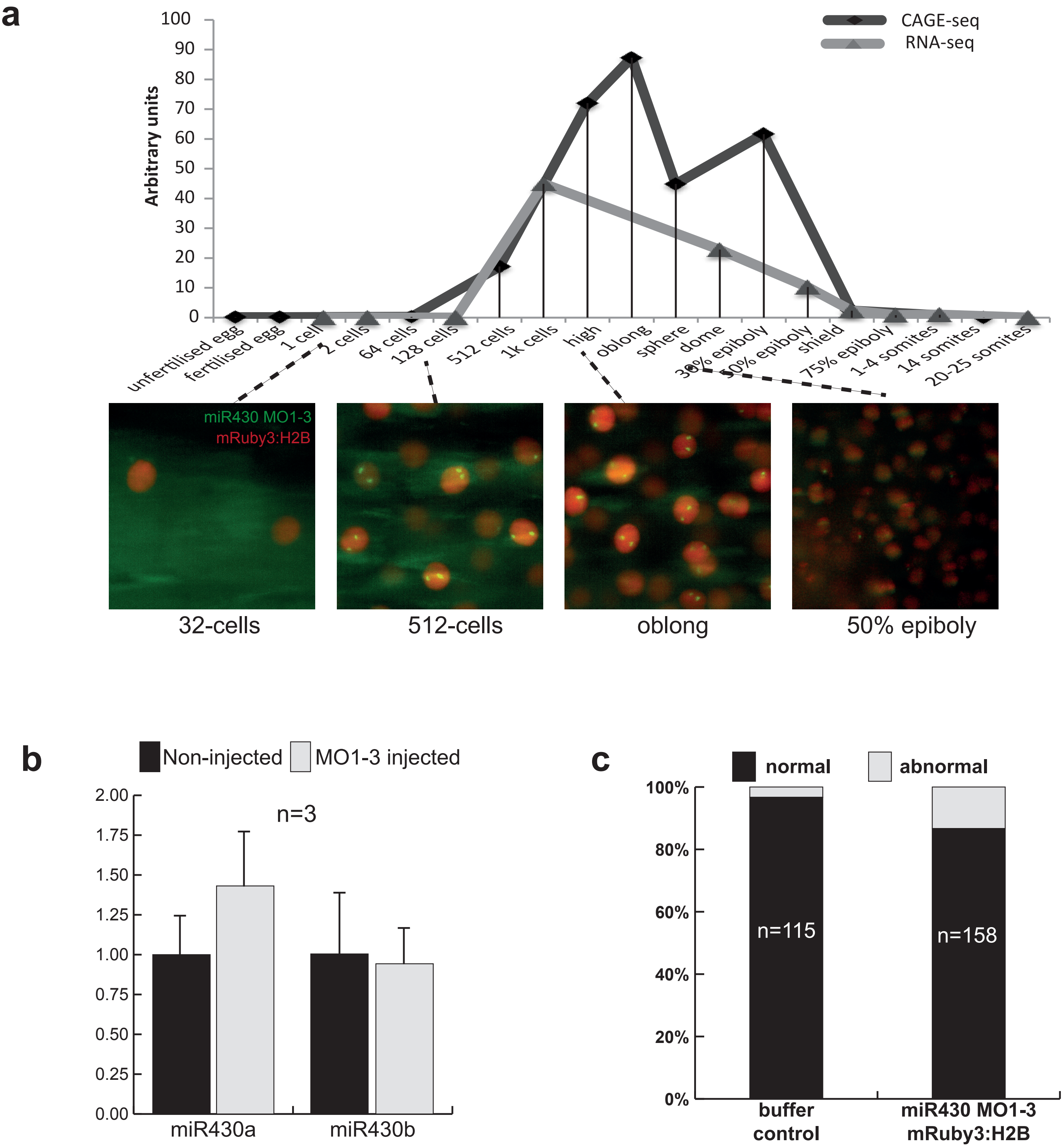

Supplementary Figure 1. Microinjected Morpholinos detect miR430 transcription without affecting miR430 miRNA production or embryo development

(a) Expression of miR430 during development detected by genome wide transcriptome assays (CAGE-seq and RNA-seq) and corresponding detection of transcripts by miR430 targeting morpholinos in vivo **(b)** Bar chart showing comparison of levels of the mature miR430a and miR430b RNA levels assayed by droplet digital PCR between non-injected control and miR430 targeting MOs, injected embryos at sphere stage. The values are mean from 3 biological repeats and normalized to the non-injected control. The error bars represent standard error of proportion. **(c)** Bar chart showing the proportion of normal and abnormal developing embryos injected with miR430 targeting MOs and mRuby3:H2B fusion protein or buffer control, assessed for normal development at 48 hpf. Source data are provided as a Source Data file.

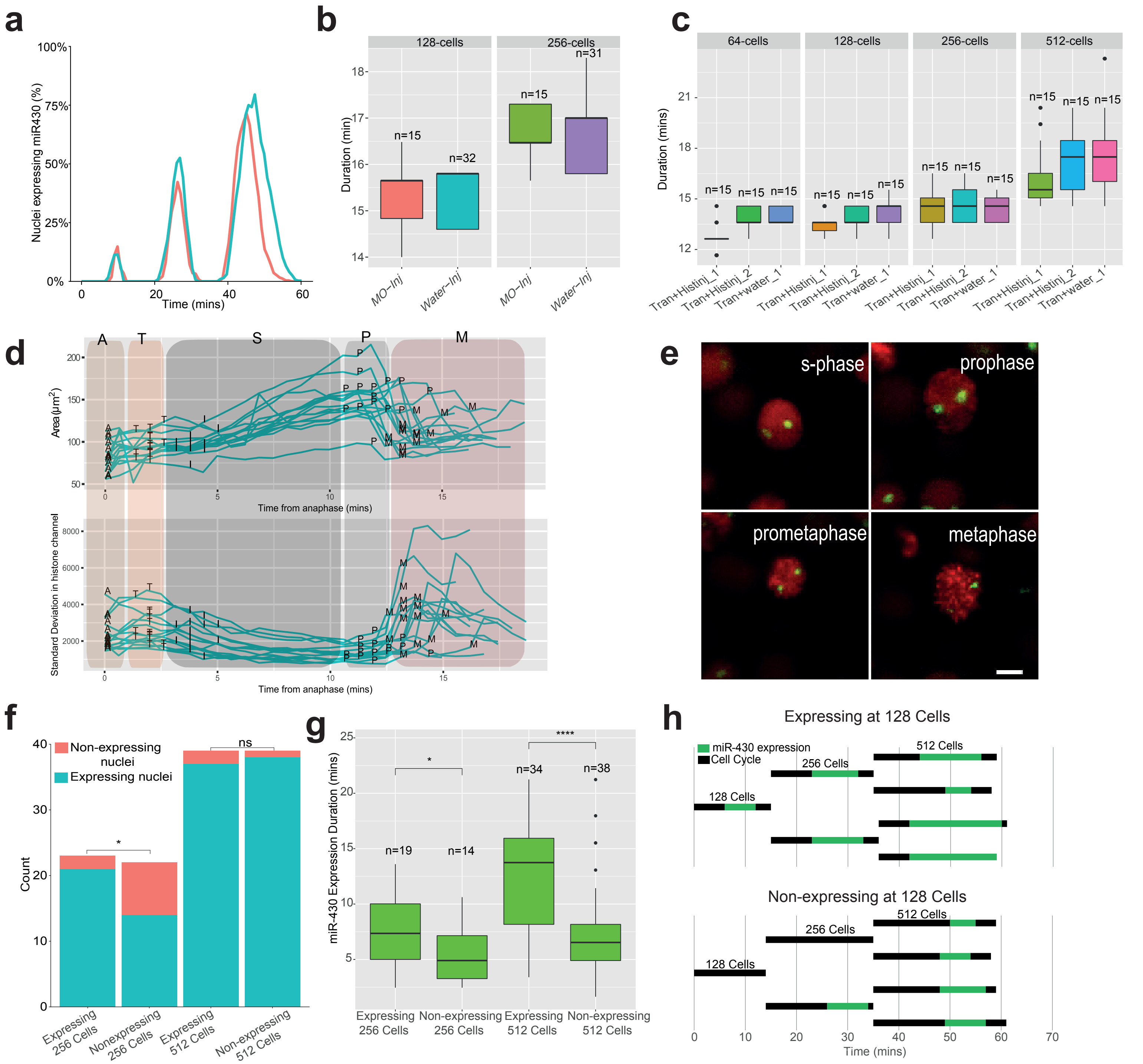

Supplementary Figure 2. Monitoring the relationship between cleavage stage cell cycles and miR430 transcript detection
(a) Percentage of visible nuclei showing miR430 transcription focus expression over time, based on maximum intensity projection lightsheet microscopy data. Data aligned at the highest point of the 64-cell stage peak. Each coloured line represents a single embryo. **(b)** Box plot comparison of cell cycle durations between morpholino injected and water injected embryos to assess the impact of morpholino on the cell cycle. Each box shows duration data from different cells within a single embryo. Embryonic stages denoted in grey headers. **(c)** Cell cycle lengths measured from anaphase to anaphase, based on lightsheet maximum intensity projection data. 'Tran' denotes transgenic, 'Histinj' denotes histone injection group. Each box shows duration data from different cells within a single embryo. Embryonic stages denoted in grey headers. **(d)** Cell cycle reference chart constructed from cell cycle analysis showing characteristic trends and features explored to help inform cell cycle segmentation. Individual lines represent imaging parameters from 2D ROI's generated around 256-cell stage nuclei from lightsheet maximum intensity projection data. Annotations on lines denote manually labelled cell cycle phase transition points, based off comparisons with reference library. Overlaid coloured boxes show overall segmentation into cell cycle phases based off common features in the line graph. **(e)** Example of a nucleus, labeled by mRuby3:H2B (red) showing miR-430 signal (green) duruing different phases of the cell cycle. **(f)** Comparison of miR-430 expression duration at different embryonic stages from different lineage groups, **(g)** counts of expressing and non-expressing nuclei which showed miR-430 expression, per cell cycle stage from each lineage group (lineage groups are based on whether the parent cell showed miR-430 expression at 128 cell stage) **(h)** Lineage trees showing a lineage where the parent nucleus showed miR-430 expression (green section of bar) at 128 cell stage (top) and the other where the parent nucleus was not expressing at 128 cell stage (bottom); each bar represents an individual nucleus from the lineage, and how long its cell cycle persisted for. On the box plots (**b, c, g**) Middle line equals median, whiskers nearest hinge ± 1.5 * interquartile range. Abbreviations: A, anaphase; T, telophase; S, S-phase; P, prophase; M metaphase. Source data for **a-d,f-g** are provided as a Source Data file.

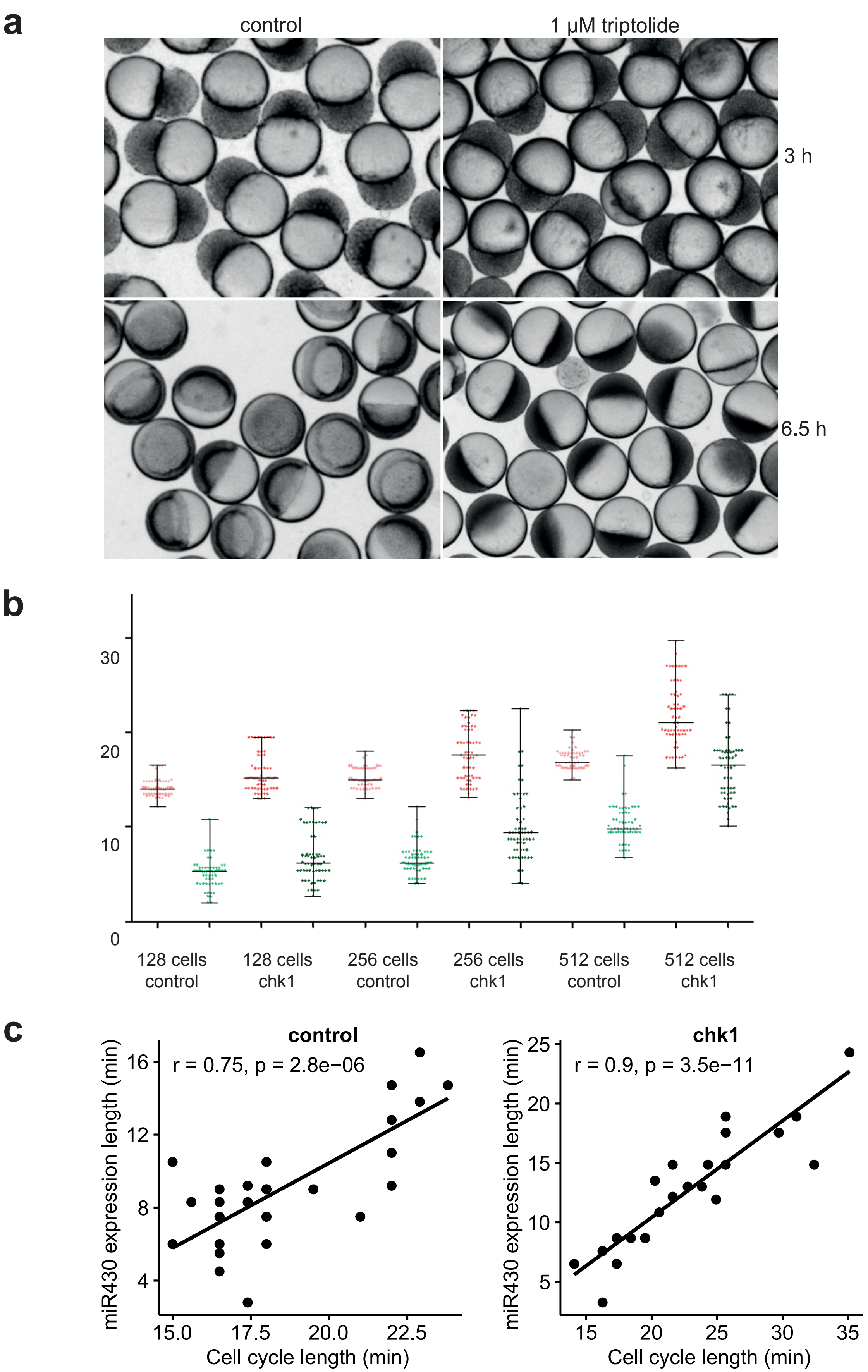

Supplementary Figure 3. Relationship between cell cycle and transcription

(a) Brightfield images of embryos treated with triptolide (n=60) as compared to control (n=65) at times denoted by hours post fertilisation (h). **(b)** Relationship between the length of cell cycle measured by mRuby3:H2B (red) and miR430 activity measured by morpholino signal (green) in control and chk1 mRNA injected embryos. Dots represent individual nuclei analysed from the same four embryos shown in Fig. 3f, 15 nuclei each at stages as indicated below the charts. Whiskers show max and min values, the middle line represents the median. **(c)** Correlation between cell cycle length and miR430 expression length in control (left) and chk1 mRNA injected embryos at 512-cells stage. The Pearson correlation coefficient (r) and the corresponding p-value (p) are shown on each plot. Data from 29 cells (two embryos) for each group were analysed. Source data for **b,c** are provided as a Source Data file.

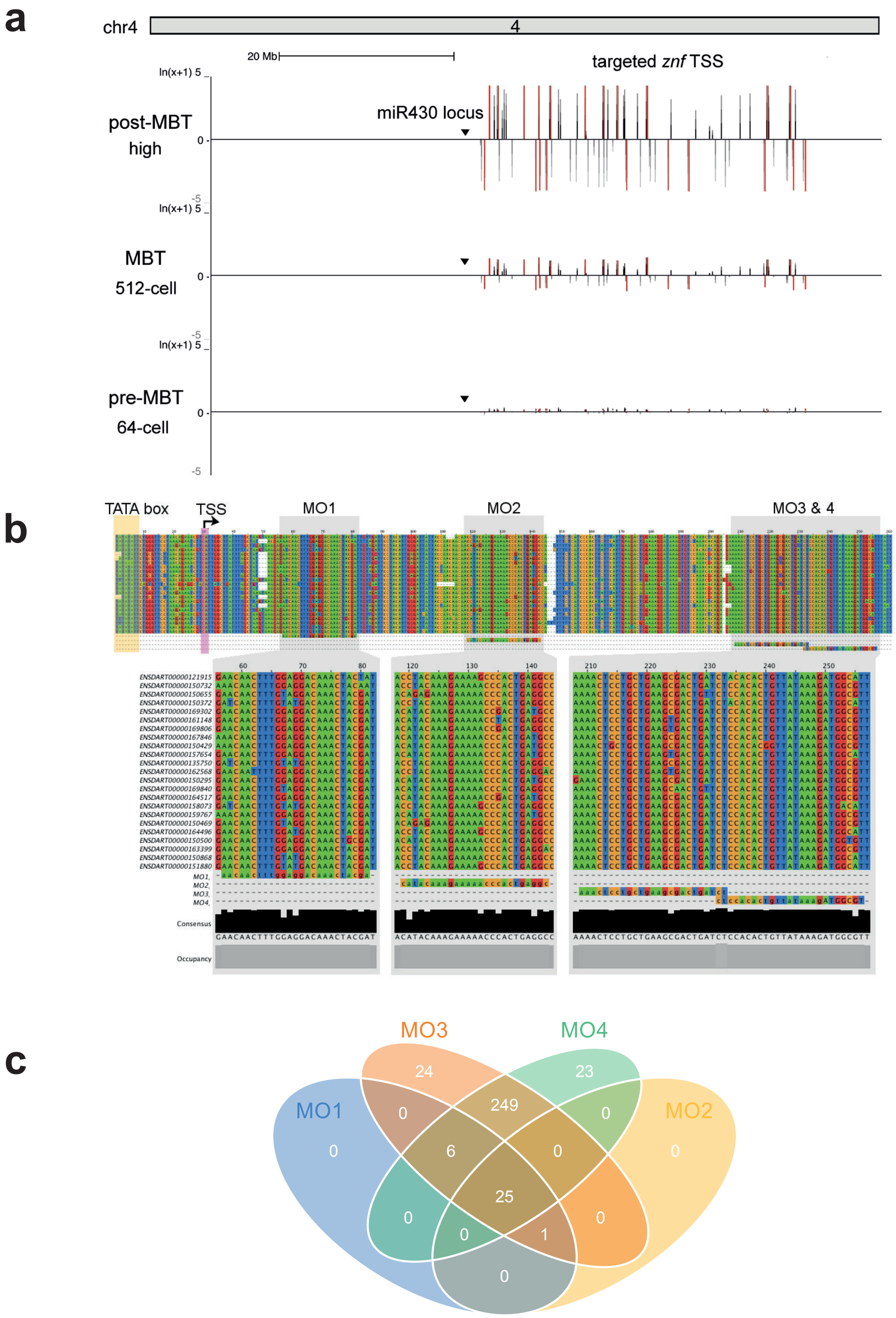

Supplementary Figure 4. Mapping, transcriptional activity and targeting of a znf gene cluster on chromosome 4 by morpholinos

(a) Locations of zinc finger genes on chromosome 4 with CAGE-seq peaks demonstrating expression in TPM (tag per million). Highly conserved set of genes which were used to design targeting MOs are highlighted in red. Location of miR430 gene cluster indicated with arrow. **(b)** Sequence alignment of highly expressed znf genes used for design of targeting MOs and location of MO sequences are shown. **(c)** Venn diagram shows single-counted intersections between znf targeting MOs and all potentially targeted znf genes by each MO with up to 4 mismatches (for mismatch details see Methods). Source data for **c** are provided as a Source Data file.

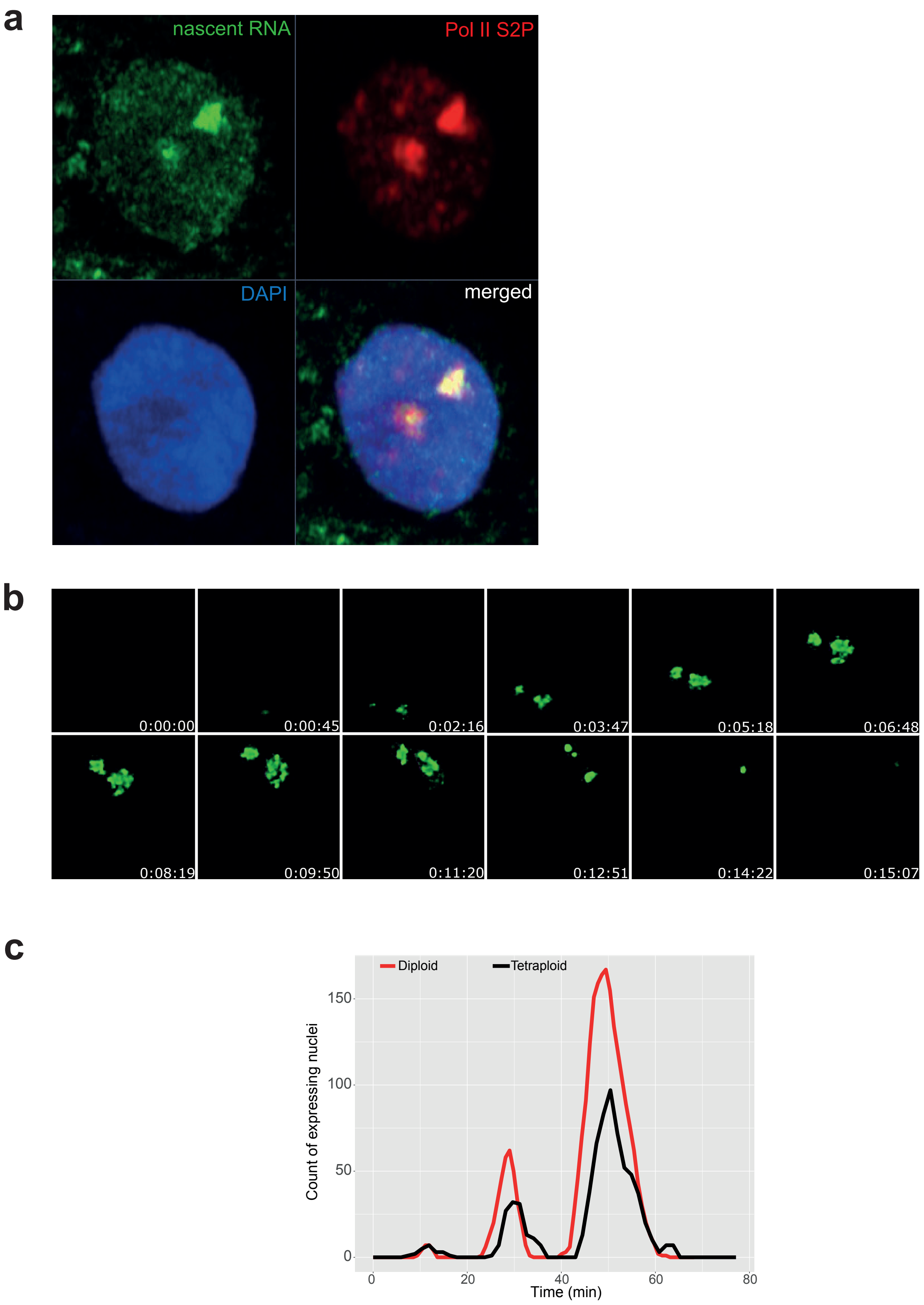

Supplementary Figure 5. Spatio-temporal features and mitotic chromosome organisation of the miR430 transcription compartment in cleavage stages

(a) Maximum intensity projection of an S phase nucleus at 256 cell stage in EU an injected embryo used for RNA pol II Ser2P immunohistochemistry. EU labelled nascent RNA (green), Pol II Ser2P (red), and DAPI (blue) are shown (data example from 49 nuclei n=6 embryos). **(b)** miR430 compartment dynamics in a single cell cycle at 512-cells stage. Timelapse taken from Suppl. Movie 4 shows the evolution of miR430 transcription foci from a single nucleus, taken from a single channel Airyscan dataset. Video timestamps inlaid in panels indicates minutes since start of signal detection within the cell cycle. **(c)** Comparison of the number of miR430 transcription foci in nuclei between a tetraploid embryo and a diploid embryo. Maximum intensity projection light sheet data were analysed, extracted using Icy bioimage to obtain focus counts. Source data for are **c** provided as a Source Data file.

a

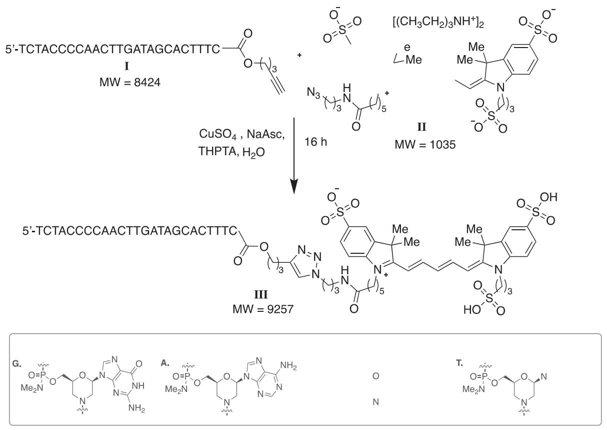

b

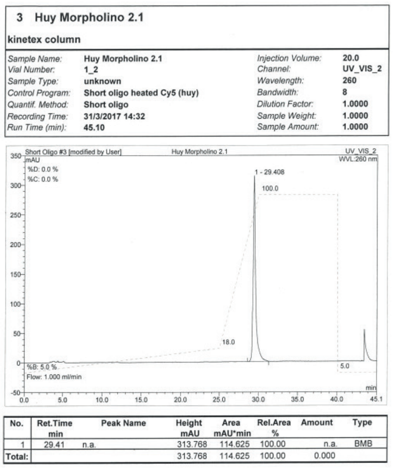

c

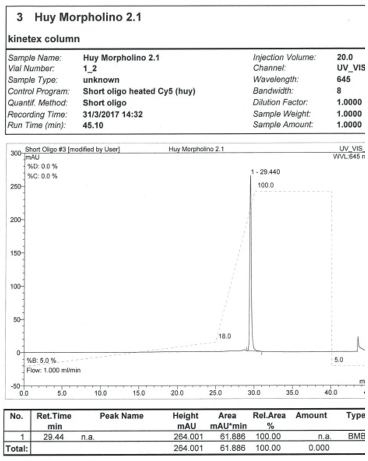

d

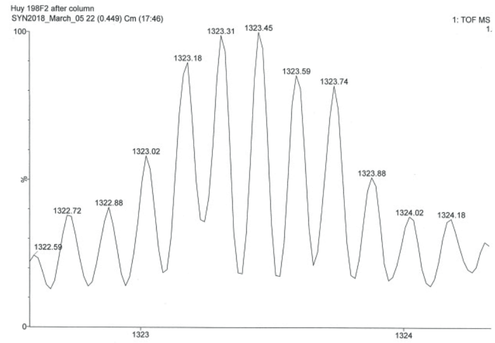

e

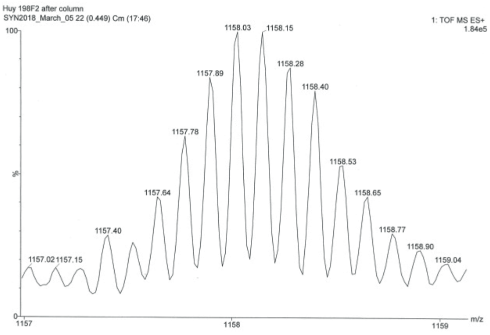

f

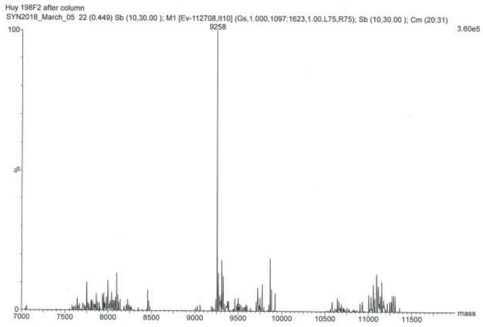

Supplementary Figure 6. Synthesis of Cy5 tagged miR430 MO4 morpholino

(a) The Copper-catalysed Azide Alkyne Cycloaddition (CuAAC) of alkyne appended morpholino sequence I with azide derivatised Cy5 II to give triazole linked Cy5 - 25mer morpholino sequence III **(b)** Analytical HPLC trace of Cy5 tagged morpholino III detecting at 260 nm; solvent A: water; solvent B: acetonitrile; stationary phase Phenomenex Kinetex; column temperature 35 ºC; flow rate 1mL/min **(c)** Analytical HPLC trace of Cy5 tagged morpholino III detecting at 645 nm; solvent A: water; solvent B: acetonitrile; stationary phase Phenomenex Kinetex; column temperature 35 ºC; flow rate 1mL/min **(d)** Raw data for ESI mass spectrum of Cy5 tagged morpholino III, m/z = 1323 with +7 charged envelope [M+7H]7+ **(e)** Raw data for ESI mass spectrum of Cy5 tagged morpholino III, m/z = 1158 with +8 charge envelopes [M+8H]8+ **(f)** Processed MAXENT ESI mass spectrometry data of Cy5 tagged morpholino III with calculated m/z of [M+H]+ 9258

Supplementary Table 1. Morpholino oligonucleotides used in this study.

**Morpholino Name**	**Sequence (5'-> 3')**	**Fluorescent Tag**
miR430_MO1	CACACGCATCTTGTTGTCTGCTGTT	3' Fluorescein
miR430_MO1_5MM	CCCACTCATATTGTTGTATGCTTTT	3' Fluorescein
miR430_M02	GCAGCAAGAGAAATCATTGGCAGGC	3' Fluorescein
miR430_MO3	AGCAGATTAAAACCTTGTCCGTTCA	3' Fluorescein
miR430_MO4	TCTACCCCAACTTGATAGCACTTTC	3' Fluorescein or 3' Cyanine 5
miR430_M01_SENSE	AACAGCAGACAACAAGATGCGTGTG	3' Fluorescein
miR430_M02_SENSE	GCCTGCCAATGATTTCTCTTGCTGC	3' Fluorescein
miR430_M03_SENSE	TGAACGGACAAGGTTTTAATCTGCT	3' Fluorescein
ZNFs_MO1	TCGTAGTTTGTCCTCCAAAGTTGTT	3' Fluorescein
ZNFs_MO1_6MM	TCTTAATTTTTCATCAAAATTTATT	3' Fluorescein
ZNFs_MO2	GCCTCAGTGGGTTTTTCTTTGTATG	3' Fluorescein
ZNFS_MO3	AGATCAGTCGCTTCAGCAGGAGTTT	3' Fluorescein
ZNFs_MO3/MO4_6MM	TAATAGTATAGAAATCAATCGTTTC	3' Fluorescein
ZNFs_MO4	ACGCCATCTTTATAACAGTGTGGAG	3' Fluorescein
ZNFs_MO4_6MM	ACTCCATATTTGTAAAAGTTTGTAG	3' Fluorescein
rRNA_5'ETS_MO1	TGCACTTAGACTTGCATGGCTTAAT	3' Fluorescein
