# Supplementary material for: A cell cycle-coordinated Polymerase II transcription compartment encompasses gene expression before global genome activation
Source: Nat Commun. 2019 Feb 11;10:691. doi: 10.1038/s41467-019-08487-5 (PMC6370886; doi:10.1038/s41467-019-08487-5)
Supplement: Supplementary file 2 — Description of Additional Supplementary Information [file 41467_2019_8487_MOESM2_ESM.pdf]

Description of Additional Supplementary Files

File Name: Supplementary Movie 1
Description: shows time-lapse of embryo injected with combination miR430 targeting MOs and mRuby3:H2B fusion protein and imaged on a lightsheet microscope from 128 cells stage. Frames were acquired every 63s.

File Name: Supplementary Movie 2
Description: shows time-lapse of Tg(Xla.Eef1a1:h2b-mRFP1) embryo injected with miR430 targeting MOs and imaged on a lightsheet microscope from 32-cell stage. Frames were acquired every 51s.

File Name: Supplementary Movie 3
Description: shows time-lapse of embryo injected with combination miR430 (red) and znf genes (green) targeting MOs and imaged on a Zeiss 880 Confocal microscope with Fast Airyscan mode from 128-cell stage. Frames were acquired every 158s.

File Name: Supplementary Movie 4
Description: Temporal dynamics of miR430 transcription compartment within a cell cycle. Animation shows growth and decay of a pair of 512- cell stage transcription foci, from the same nucleus. Green indicates miR430 morpholino signal. Data subsetted and recorded in Icy Bioimage 3D VTK viewer. Time stamps indicates time from first detection of miR430 signal in second and minutes.

File Name: Supplementary Movie 5
Description: 3D rendering of nucleus and miR430 transcription compartment. Nucleus render (beige) from 1K-cells stage embryo was produced using the red channel (mRuby3 H2B). The ROI of the miR430 transcription compartment was isolated from the green channel (miR430 MOs). Video was rendered and recorded in Icy Bioimage. Quadrant was cut away to expose miR430 transcription compartment.

File Name: Supplementary Movie 6
Description: Expression of miR430 in tetraploid embryo. Tetraploid embryo injected with miR430 targeting MOs were imaged on a Zeiss 880 Confocal microscope with Fast Airyscan mode from 256-cell stage. Frames were acquired every 43s.

File Name: Supplementary Movie 7
Description: 3D rendering of Immunohistochemistry of Pol II S2P (red) and nascent RNA detection (green) by ethynyl-uridine (EU) incorporation and Alexa Fluor 488 tagging at 256-cell stage. The nucleus is stained with DAPI (blue). The rendering was performed with Zeiss ZEN blue software.
