# Supplementary material for: A cell cycle-coordinated Polymerase II transcription compartment encompasses gene expression before global genome activation
Source: Nat Commun. 2019 Feb 11;10:691. doi: 10.1038/s41467-019-08487-5 (PMC6370886; doi:10.1038/s41467-019-08487-5)
Supplement: Supplementary file 10 — Reporting Summary [file 41467_2019_8487_MOESM10_ESM.pdf]

nature research

Corresponding author(s): Ferenc Müller

Reporting Summary

Nature Research wishes to improve the reproducibility of the work that we publish. This form provides structure for consistency and transparency in reporting. For further information on Nature Research policies, see Authors & Referees and the Editorial Policy Checklist.

Statistical parameters

When statistical analyses are reported, confirm that the following items are present in the relevant location (e.g. figure legend, table legend, main text, or Methods section).

n/a	Confirmed	
☐	☒	The exact sample size (*n*) for each experimental group/condition, given as a discrete number and unit of measurement
☐	☒	An indication of whether measurements were taken from distinct samples or whether the same sample was measured repeatedly
☐	☒	The statistical test(s) used AND whether they are one- or two-sided<br>*Only common tests should be described solely by name; describe more complex techniques in the Methods section.*
☒	☐	A description of all covariates tested
☐	☒	A description of any assumptions or corrections, such as tests of normality and adjustment for multiple comparisons
☒	☐	A full description of the statistics including central tendency (e.g. means) or other basic estimates (e.g. regression coefficient) AND variation (e.g. standard deviation) or associated estimates of uncertainty (e.g. confidence intervals)
☐	☒	For null hypothesis testing, the test statistic (e.g. *F*, *t*, *r*) with confidence intervals, effect sizes, degrees of freedom and *P* value noted<br>*Give P values as exact values whenever suitable.*
☒	☐	For Bayesian analysis, information on the choice of priors and Markov chain Monte Carlo settings
☒	☐	For hierarchical and complex designs, identification of the appropriate level for tests and full reporting of outcomes
☐	☒	Estimates of effect sizes (e.g. Cohen's *d*, Pearson's *r*), indicating how they were calculated
☐	☒	Clearly defined error bars<br>*State explicitly what error bars represent (e.g. SD, SE, CI)*

Our web collection on statistics for biologists may be useful.

Software and code

Policy information about availability of computer code

Data collection	N/A
Data analysis	Imaging data were analyzed by using publicly available softwares, which are cited in the paper.

For manuscripts utilizing custom algorithms or software that are central to the research but not yet described in published literature, software must be made available to editors/reviewers upon request. We strongly encourage code deposition in a community repository (e.g. GitHub). See the Nature Research guidelines for submitting code & software for further information.

Data

Policy information about availability of data

All manuscripts must include a data availability statement. This statement should provide the following information, where applicable:
- Accession codes, unique identifiers, or web links for publicly available datasets
- A list of figures that have associated raw data
- A description of any restrictions on data availability

Availability of movie data used for analyses are listed in Suppl. Table 2.

Field-specific reporting

Please select the best fit for your research. If you are not sure, read the appropriate sections before making your selection.

☒ Life sciences ☐ Behavioural & social sciences ☐ Ecological, evolutionary & environmental sciences

For a reference copy of the document with all sections, see nature.com/authors/policies/ReportingSummary-flat.pdf

Life sciences study design

All studies must disclose on these points even when the disclosure is negative.

Sample size	No statistical methods were used to predetermine sample size.
Data exclusions	No data were excluded from the analyses.
Replication	All presented experiments are based on data, which included more than one replicate sample. Major conclsuions were drawn from more than one orthogonal approaches.
Randomization	No randomization was required in the study.
Blinding	No blinding was required in the study.

Reporting for specific materials, systems and methods

Materials & experimental systems

n/a	Involved in the study
☒	☐ Unique biological materials
☐	☒ Antibodies
☒	☐ Eukaryotic cell lines
☒	☐ Palaeontology
☐	☒ Animals and other organisms
☒	☐ Human research participants

Methods

n/a	Involved in the study
☒	☐ ChIP-seq
☐	☒ Flow cytometry
☒	☐ MRI-based neuroimaging

Antibodies

Antibodies used	Anti-RNA polymerase II (phospho S2), catalogue # ab5095, lot # GR231750-2, Abcam at 1:400 dilution<br>Pol II S2p monoclonal antibody, catalogue # C15200005, lot # 001-11, Diagenode at 1:400 dilution<br>Anti-Histone H3 (di methyl K79) antibody, catalogue # ab3594, lot # 803369, Abcam at 1:1000 dilution
Validation	Only previously validated commercially available antibodies were used in the study.

Animals and other organisms

Policy information about studies involving animals; ARRIVE guidelines recommended for reporting animal research

Laboratory animals	Only early life form of zebrafish embryos up to the free feeding stage (5 days post fertilization) were used in this study. Zebrafish embryos up to the free feeding stage are not considered as animal by law in the UK or the EU and are not subjected to animal experimentation regulations. Transgenic zebrafish embryos were used under the Home Office project license 40/3681 and P51AB7F76 assigned to the University of Birmingham, UK.
Wild animals	N/A
Field-collected samples	The study did not collect samples collected in the field.

Flow Cytometry

Plots

Confirm that:

☒ The axis labels state the marker and fluorochrome used (e.g. CD4-FITC).

☒ The axis scales are clearly visible. Include numbers along axes only for bottom left plot of group (a 'group' is an analysis of identical markers).

☒ All plots are contour plots with outliers or pseudocolor plots.

☒ A numerical value for number of cells or percentage (with statistics) is provided.

Methodology

Sample preparation	Embryos were collected at the 12-somite stage, dissociated with PBS-based enzyme-free cell dissociation buffer (Gibco), washed with PBS and subjected to propidium iodide DNA content analysis following manufacturer’s conditions (Invitrogen).
Instrument	Beckman Coulter CyAn ADP
Software	Summit 4.4 software was used
Cell population abundance	The entire viable cell population is shown for each population
Gating strategy	A standard PI gating strategy was used as described in:<br>Davies 2012. Propidium iodide staining of cells to assess DNA cell cycle. Abcam online protocols. (Accessed 7.12.18). https://www.med.cam.ac.uk/wp-content/uploads/2012/10/pi.pdf<br><br>As the gating strategy used is a standard ,extensively published, protocol we determined it unnecessary to include an illustrative supplementary figure.

☐ Tick this box to confirm that a figure exemplifying the gating strategy is provided in the Supplementary Information.
